# New Insights into the Evolution of *Wolbachia* Infections in Filarial Nematodes Inferred from a Large Range of Screened Species

**DOI:** 10.1371/journal.pone.0020843

**Published:** 2011-06-22

**Authors:** Emanuele Ferri, Odile Bain, Michela Barbuto, Coralie Martin, Nathan Lo, Shigehiko Uni, Frederic Landmann, Sara G. Baccei, Ricardo Guerrero, Sueli de Souza Lima, Claudio Bandi, Samuel Wanji, Moustapha Diagne, Maurizio Casiraghi

**Affiliations:** 1 Dipartimento di Biotecnologie e Bioscienze, Università degli Studi di Milano Bicocca, Milano, Italy; 2 Parasitologie Comparée UMR 7205 CNRS & UMR 7245 CNRS, Muséum National d'Histoire Naturelle, Paris, France; 3 School of Biological Sciences, University of Sydney, Sidney, Australia; 4 Department of Medical Zoology, Osaka City University Medical School, Osaka, Japan; 5 Department of Molecular, Cell and Developmental Biology, University of California Santa Cruz, Santa Cruz, California, United States of America; 6 Instituto de Zoologia Tropical, Universidad Central de Venezuela, Caracas, Venezuela; 7 Departamento de Zoologia, Universidade Federal de Juiz de Fora, Minas Geraes, Brasil; 8 Dipartimento di Patologia Animale, Igiene e Sanità Pubblica Veterinaria, Università degli Studi di Milano, Milano, Italy; 9 Research Foundation in Tropical Diseases and Environment, Buea, Cameroun; 10 Département de Biologie Animale, Université Cheikh Anta Diop de Dakar, Dakar, Sénégal; Agency for Science, Technology and Research - Singapore Immunology Network, Singapore

## Abstract

**Background:**

*Wolbachia* are intriguing symbiotic endobacteria with a peculiar host range that includes arthropods and a single nematode family, the Onchocercidae encompassing agents of filariases. This raises the question of the origin of infection in filariae. *Wolbachia* infect the female germline and the hypodermis. Some evidences lead to the theory that *Wolbachia* act as mutualist and coevolved with filariae from one infection event: their removal sterilizes female filariae; all the specimens of a positive species are infected; *Wolbachia* are vertically inherited; a few species lost the symbiont. However, most data on *Wolbachia* and filaria relationships derive from studies on few species of Onchocercinae and Dirofilariinae, from mammals.

**Methodology/Principal Findings:**

We investigated the *Wolbachia* distribution testing 35 filarial species, including 28 species and 7 genera and/or subgenera newly screened, using PCR, immunohistochemical staining, whole mount fluorescent analysis, and cocladogenesis analysis. (i) Among the newly screened Onchocercinae from mammals eight species harbour *Wolbachia* but for some of them, bacteria are absent in the hypodermis, or in variable density. (ii) *Wolbachia* are not detected in the pathological model *Monanema martini* and in 8, upon 9, species of *Cercopithifilaria*. (iii) Supergroup F *Wolbachia* is identified in two newly screened *Mansonella* species and in *Cercopithifilaria japonica*. (iv) Type F *Wolbachia* infect the intestinal cells and somatic female genital tract. (v) Among Oswaldofilariinae, Waltonellinae and Splendidofilariinae, from saurian, anuran and bird respectively, *Wolbachia* are not detected.

**Conclusions/Significance:**

The absence of *Wolbachia* in 63% of onchocercids, notably in the ancestral Oswaldofilariinae estimated 140 mya old, the diverse tissues or specimens distribution, and a recent lateral transfer in supergroup F *Wolbachia*, modify the current view on the role and evolution of the endosymbiont and their hosts. Further genomic analyses on some of the newly sampled species are welcomed to decipher the open questions.

## Introduction

The alpha proteobacteria *Wolbachia* (Rickettsiales) are present in two distinct zoological groups: the arthropods, where they are widespread [1], and the nematodes, where they are restricted to a single but notable family of parasites, the Onchocercidae [2], [3]. They encompass the agents of human onchocerciasis and lymphatic filariases [4]. The zoological host range of *Wolbachia* raised a fundamental question on the origin of infection in the filarial nematodes [5], [6]. Investigations performed during the past fifteen years on *Wolbachia* in filarial and arthropod hosts has led to establish a rather clear and complex picture of the taxonomic status of the bacterium, its distribution and phylogeny [7]. Several distinct bacterial lineages have been called supergroups [8], and, at this date, they are all attributed to the only valid recognized species *Wolbachia pipientis*. The taxonomy of this species is quite uncertain, and in the scientific literature the genus name *Wolbachia* has been widely used as a specific name. This is taxomically incorrect, but common in microbiology (where species concept is usually complicated) and in the present work we will follow this trend until new data will be made available for a proper taxonomic restructuring [9], [10]. The supergroups are in majority distinct in arthropods and filariae: A, B, E, H, I, K are found in the arthropods; C, D and J in the nematodes [6], [8], [11]. However, the supergroup F is a relevant and very well supported exception, encompassing arthropod and filarial hosts (i.e. some insects such as termites and the human filariae of the genus *Mansonella*, [12]–[15]). Moreover, a newly discovered *Wolbachia* harboured by a plant parasitic nematode might represent a further supergroup [16], while the supergroup G [17] has been decommissioned due to the high probability of being characterised on the basis of an event of recombination [18].

Whereas the bacteria are mainly parasites in arthropods, usually acting as manipulators of reproduction [19]–[21], they are mutualistic in filariae [4], [22]. These mechanisms may be diverse, considering that *Wolbachia* is not only present in the germline but also in a somatic tissue, the hypodermis (lateral chords) of both females and males [23]–[25]. The biological studies and the *Wolbachia* genome projects [26] allowed us to suppose that the bacteria may be essential in the biosynthesis of some molecules necessary for filarial host fertility and viability, such as heme, riboflavin or nucleotide synthesis. Biosynthetic pathways are currently analyzed to determine the components of the symbiotic relationships [27]–[32]. To date, the mutualistic partnership is targeted in treatments against filariases using antibiotics [33].

The spiruroid ancestors of filariae that have been screened so far are devoid of endobacteria [5], [34]. The presence/absence of *Wolbachia* mapped on a filarial nematodes phylogenetic tree suggests that the bacteria may have possibly been acquired as a single event in the lineage leading to the onchocercid nematodes, followed by host-parasite co-evolution, assuming that *Wolbachia* in filariae was strictly vertically transmitted to the offspring through the infected female germline [3], [35]. Analysis of supergroup F is changing this view, due to the presence of *Wolbachia* from both filariae and some insects.

Another potential discrepancy with respect to the suggestion of coevolution is provided by observations of the absence of *Wolbachia* in two filarial species within the onchocercid lineage: the human parasite *Loa loa* and the rodent parasite *Acanthocheilonema viteae* [3], [36]. It has been suggested that for these host species, the endobacteria had been present but subsequently were lost during further evolution [5]. As a corollary, the loss of *Wolbachia* led to a further hypothesis that the bacterial genes essential to the host fitness might have been successfully transferred and expressed into the host genome. Although still subject of discussion [21], some support for this hypothesis derives from studies on lateral gene transfer, as shown with several insect and filarial hosts [37]. Remnants of *Wolbachia*-like gene sequences have been identified in the filarial host genomes of the endobacteria-free *L. loa* and *A. viteae,* with some of the transferred genes being transcribed [38]. The elimination of the bacteria might be an adaptive advantage because their antigens are inflammation inducers and contribute to filarial pathologies and immunological responses [39]. However, a recent study suggests that the bacteria might act as a decoy target for polynuclear neutrophils, preventing harmful effect of eosinophils on filariae [40]. Furthermore, a strain of *Wolbachia* that over-replicates in *Aedes aegypti* inhibits the development of *Brugia malayi* larvae and switches on a few important immune system genes [41]–[43]. Thus the limitation of the filarial infection may either be due to immune activation of the invertebrate host or/and the bacteria may outcompetes filariae for some metabolites.

In our previous study [5], it appeared that the number of endobacteria-free filarial species had been underestimated and that several species without *Wolbachia* detected were parasitic in lizards and frogs. Thus it was suggested that these filariae from reptiles and anurans diversified before the first bacterial invasion on the onchocercid lineage which had been tentatively dated to 110 mya [3], [6]. Indeed the origin of the Oswaldofilariinae, parasitic in crocodiles and squamates, was hypothetically dated from the late Jurassic, at the beginning of Gondwanian dislocation, 140 mya [44], [45]. However representatives of this subfamily had not yet been screened.

Until the present study, *Wolbachia* screening had been done in only about 10% of the 93 genera currently recognized in the Onchocercidae [46], [47]. This is not surprising since the recovery of filariae from connective tissues, their main localization, is not easy. Our investigation was resolutely rooted in biodiversity, expecting that the exploration of a broader range of filarial species would contribute to decipher the history of the *Wolbachia*-filaria symbiosis. The recovery of materials from wild animals from several biomes was undertaken. The first oswaldofilarine, the first splendidofilarine (a parasite of birds), and several onchocercid genera from mammals have now been screened through PCR, as well as classic immunohistochemical staining and whole mount fluorescent analysis [21].

This study confirms that *Wolbachia* are not detected until now in the filarioid species parasitic in amphibians and reptiles. Several other features have emerged from this study: i) lateral hypodermal localization of *Wolbachia* is not obligatory in bacteria-positive filarial species; ii) new somatic tissue localizations of the bacteria are observed; iii) the number of *Wolbachia*-free filarial species is greater than expected among filariae of mammals; (iv) lastly, one secondary event of *Wolbachia* infection, also well supported by a formal cocladogenesis, likely took place in filariae in supergroup F.

## Results

The screening for *Wolbachia* was performed on 35 species (Table 1; specimens detailed in Figure S1 and Tables S1, S2), of which 28 are here examined for the first time and one recently by us [48]. These were the first representatives of Oswaldofilariinae, *Piratuba scaffi* from a lizard, and of Splendidofilariinae, *Aproctella* sp. 1 from passeriforms; two more species, *Ochoterenella* sp. 1 and *O. royi,* in Waltonellinae, a subfamily restricted to anurans; five genera of Onchocercinae parasitic in mammals, a species of *Monanema*, *Mo. martini*, parasitic in African murids and used during a decade as a model for onchocerciasis because of its skin-dwelling microfilariae [49]; several species of *Cercopithifilaria*, six from ruminants, one from a bear (all from Japan), and one species from an African porcupine; *Loxodontofilaria*, with the recently described *Lo. caprini*, recovered from a Japanese caprine bovid [50]; in the genus *Mansonella*, two of the six subgenera, *Tetrapetalonema* and *Cutifilaria*, with a species each, *M. (T.) atelensis amazonae* from a monkey, and *M. (Cu.) perforata* from a cervid; and the recently studied species of *Litomosa* from a South African bat (reported in [48].

**Table 1:** The 35 species of filariae included in this study, their hosts and collection place.

N°	Subfamily	Genus (subgenus)	Species	Host	Collection place
1	**Oswaldofilariinae**	***Piratuba***	***scaffi*** Bain, 1974	*Ameiva ameiva* (Lizard jungle runner)	Venezuela
2	Waltonellinae	*Ochoterenella*	***royi*** Bain, Kim & Petit, 1979	*Bufo marinus* (Cane toad)	Venezuela
3			**sp. 1**	*Phyllomedusa bicolor* (Giant leaf frog)	French Guyana
4	Setariinae	*Setaria*	*digitata* (Linstow, 1906)	*Bos taurus* (cattle)	Japan
5			***tundra***	*Capreolus capreolus* (Roe-deer)	France
6			**sp. 1**	*Redunca fulvorufula adamaue* (Mountain Reedbuck)	Cameroon
7			**sp. 2**	*Equus burchelli* (Plains zebra)	Namibia
8			**sp. 3**	*Oryx gazella* (Gemsbok)	Namibia
9	Dirofilariinae	*Foleyella*	***candezei*** (Fraipont, 1882)	*Agama agama* (Rainbow agama)	Togo
10		*Dirofilaria*	*repens* Railliet and Henry, 1911	*Homo sapiens* (Human)	Italy
11		*Loa*	*loa* (Cobbold, 1864)	*Homo sapiens* (Human)	Cameroon
12	Onchocercinae	***Cercopithifilaria***	***bulboidea*** Uni & Bain, 2001	*Nemorhaedus crispus* (Japanese serow)	Japan
13			***crassa*** Uni, Bain & Takaoka, 2002	*Cervus nippon* (Sika deer)	Japan
15			***japonica*** (Uni, 1983)	*Ursus thibetanus* (Japanese black bear)	Japan
14			***longa*** Uni, Bain & Takaoka, 2002	*Cervus nippon* (Sika deer)	Japan
16			***minuta*** Uni & Bain, 2001	*Nemorhaedus crispus* (Japanese serow)	Japan
17			***multicauda*** Uni & Bain, 2001	*Nemorhaedus crispus* (Japanese serow)	Japan
18			***roussilhoni*** Bain, Petit & Chabaud, 1986	*Atherurus africanus* (Brush-tailed porcupine)	Gabon
19			***shohoi*** Uni, Suzuki & Katsumi, 1998	*Nemorhaedus crispus* (Japanese serow)	Japan
20			***tumidicervicata*** Uni & Bain, 2001	*Nemorhaedus crispus* (Japanese serow)	Japan
21		*Dipetalonema*	*gracile* (Rudolphi, 1809)	*Cebus olivaceus* (Capuchin monkey)	Venezuela
22		*Litomosa* *	*chiropterorum* Ortlepp, 1932	*Miniopterus natalensis* (Natal Long-Fingered Bat)	South Africa
23		*Litomosoides*	*sigmodontis* Chandler, 1931	*Meriones unguiculatus* (Jird)	MNHN strain
				*Sigmodon hispidus* (Hispid cotton rat)	Venezuela
24			***taylori*** Guerrero & Bain, 2011	*Nectomys palmipes* (Water Nectomys)	Venezuela
25			*yutajensis* Guerrero, Martin & Bain, 2003	*Pteronotus parnellii* (Mustached bat)	Venezuela
26		***Loxodontofilaria***	***caprini*** Uni & Bain, 2006	*Capricornis crispus* (Japanese serow)	Japan
27		*Mansonella * ***(Cutifilaria)***	***perforata*** Uni, Bain & Takaoka, 2004	*Cervus nippon* (Sika deer)	Japan
28		*Mansonella * ***(Tetrapetalonema)***	***atelensis amazonae***	*Cebus olivaceus* (Capuchin monkey)	Venezuela
29		***Monanema***	***martini*** Bain, Bartlett & Petit, 1986	*Arvicanthis niloticus* (African Grass Rat)	Senegal
30		*Onchocerca*	***dewittei japonica*** Uni, Bain & Takaoka, 2001	*Sus scrofa leucomystax* (Japanese wild boar)	Japan
31			***eberhardi*** Uni & Bain, 2007	*Cervus nippon* (Sika deer)	Japan
32			***skrjabini*** Ruklyadev, 1964	*Cervus nippon* (Sika deer)	Japan
				*Nemorhaedus crispus* (Japanese serow)	Japan
33			***suzukii*** Yagi, Bain & Shoho, 1994	*Nemorhaedus crispus* (Japanese serow)	Japan
34			*volvulus* (Leuckart, 1893)	*Homo sapiens* (Human)	Cameroon
35	**Splendidofilarinae**	***Aproctella***	**sp. 1**	*Turdus rufiventris* (Rufous-bellied Thrush)	Brasil
				*Saltator similis* (Green-winged Saltator)	Brasil

The presentation follows the classification of Onchocercidae by Anderson & Bain (2009).

N°: number attributed to species in this study. Species, genera, subgenera and subfamilies screened for the first time are in bold characters. * Species screened in Junker et al., 2009.

**Figure 1:**
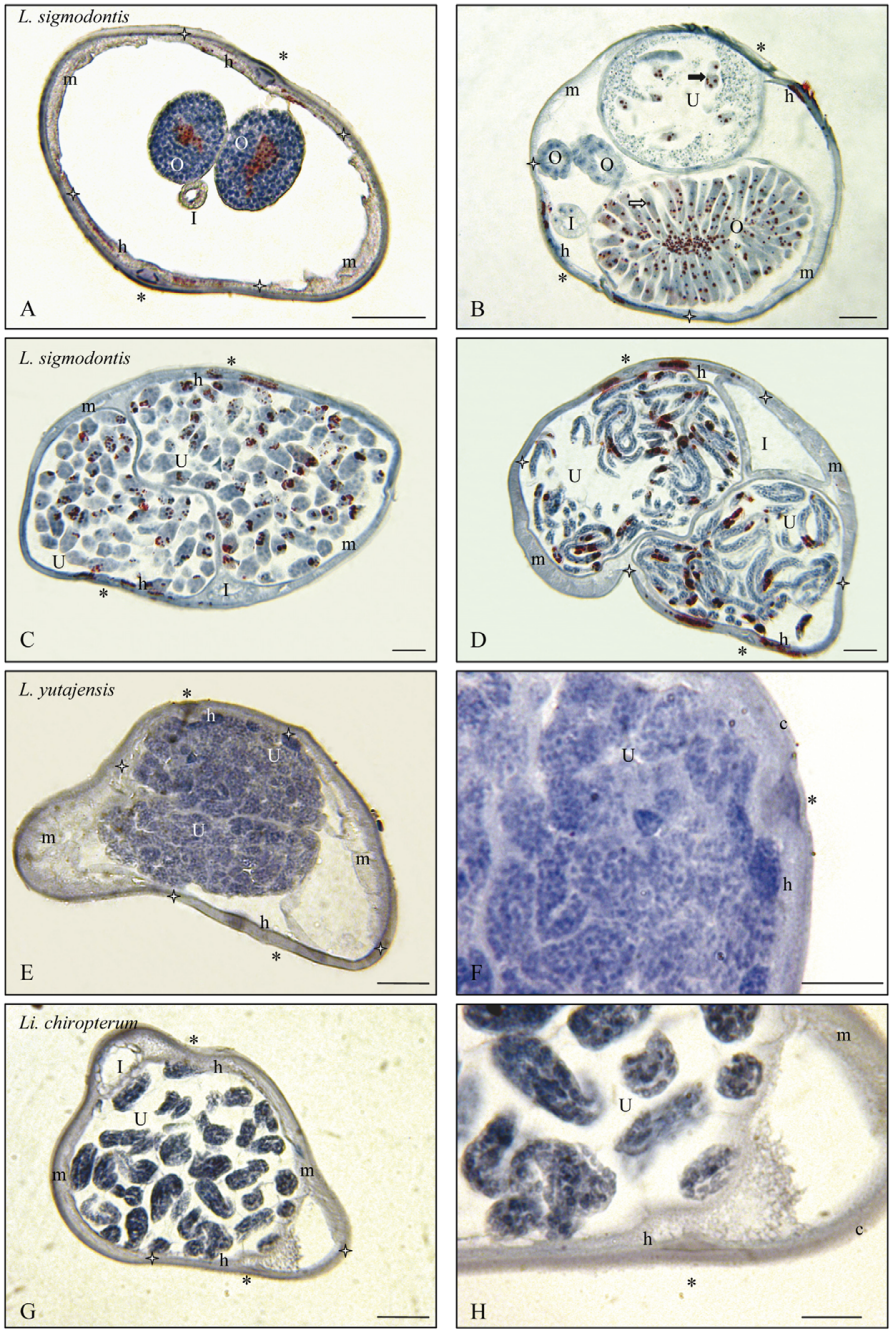
*Wolbachia* immunostaining in the genera *Litomosoides* (L.) and *Litomosa* (Li.). Transverse sections of gravid females of three species. A–D. *Litomosoides sigmodontis*, laboratory strain. A. Cuticle, lateral crests and muscles thin; hypodermal lateral chords Wb+; female germline Wb+, as seen in the rachis of the distal region of ovaries. B. Less distal region of an ovary Wb+, uterus with ova/eggs Wb+ and spermatozoa Wb–. C. Uteri with many divided eggs Wb+. D. Uteri with many microfilariae Wb+. E. *Litomosoides yutajensis*: cuticle and lateral hypodermis thin; lateral crests and muscles obvious; hypodermal lateral chords Wb–, germ line Wb–, as seen with intra-uterine divided eggs. F. Detail. G. *Litomosa chiropterorum*: muscles, lateral cuticular crests and hypodermis well distinct; hypodermal lateral chords and germ line Wb–. H. Detail of uteri with microfilarioid eggs Wb–. I, Intestine; O, Ovary; R, Rachis of ovary; U, Uterus; c, cuticle; h, hypodermal lateral chords demarcated by white stars; m, muscles; *, lateral plan; white arrow, oocytes; full arrow, ovulae. Scale bars: F & H, 10 µm; others, 25 µm. Sections were stained with a rabbit polyclonal antiserum against *Wolbachia* Surface Protein (WSP) of *Brugia pahangi Wolbachia* (Wol-Bp-WSP, dilution 1∶2000).

**Figure 2 pone-0020843-g002:**
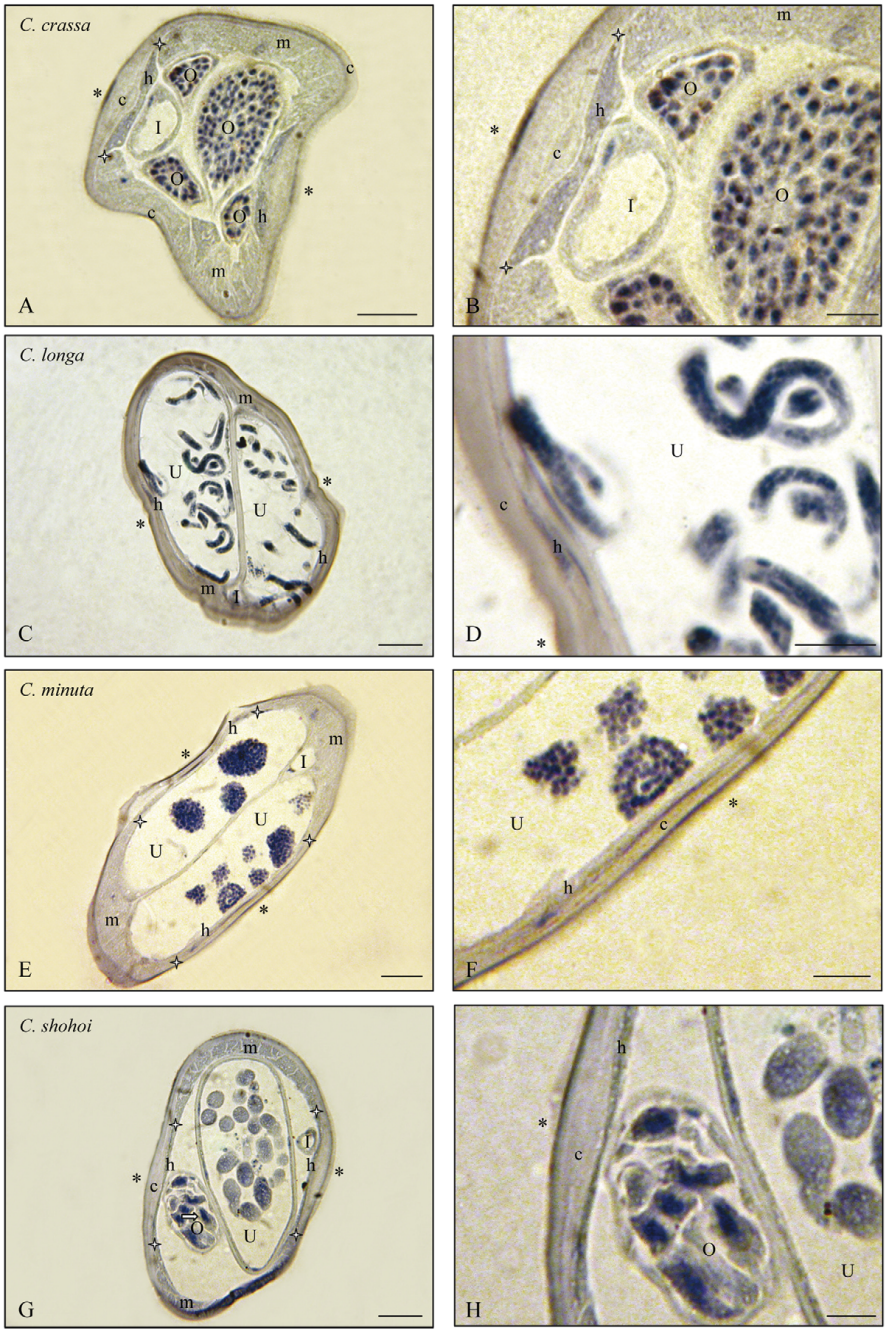
*Wolbachia* immunostaining in the genera *Onchocerca* (O.) and *Loxodontofilaria* (Lo). Transverse sections of females of four species of filariae with Wb+ germ line, at two magnifications. A. *Onchocerca dewittei japonica*: cuticle thick, lateral crest not marked, hypodermal chords well delineated and Wb–, large section of uterus and small sections of ovaries Wb+. B: Detail of lateral hypodermal chord Wb– and ovary Wb+. C: Detail of intra-uterine ova and ovary Wb+. D. *Onchocerca eberhardi*: thick cuticle with few ridges, thin muscles and thinner Wb+ lateral hypodermis; uteri with a few sections of divided eggs Wb+. E. Detail. F. *Onchocerca skrjabini*: cuticle very thick, with particular crescent-like layer forming the lateral crest, very thin lateral hypodermis Wb+ and empty uteri. G. Detail. H. *Loxodontofilaria caprini*: cuticle thick, well-developed muscle cells, thinner lateral chords Wb–; two uteri, one empty, one with Wb+ ova. I. Detail. I, Intestine; O, Ovary; U, Uterus; c, cuticle; h, hypodermal lateral chords delimitated by white stars; m, muscles; *, lateral plan; thin arrow, detail of *Wolbachia*. Scale bars: A,D,H, 50 µm; B,C,E,G,I, 25 µm. For staining, see Fig. 1.

**Figure 3 pone-0020843-g003:**
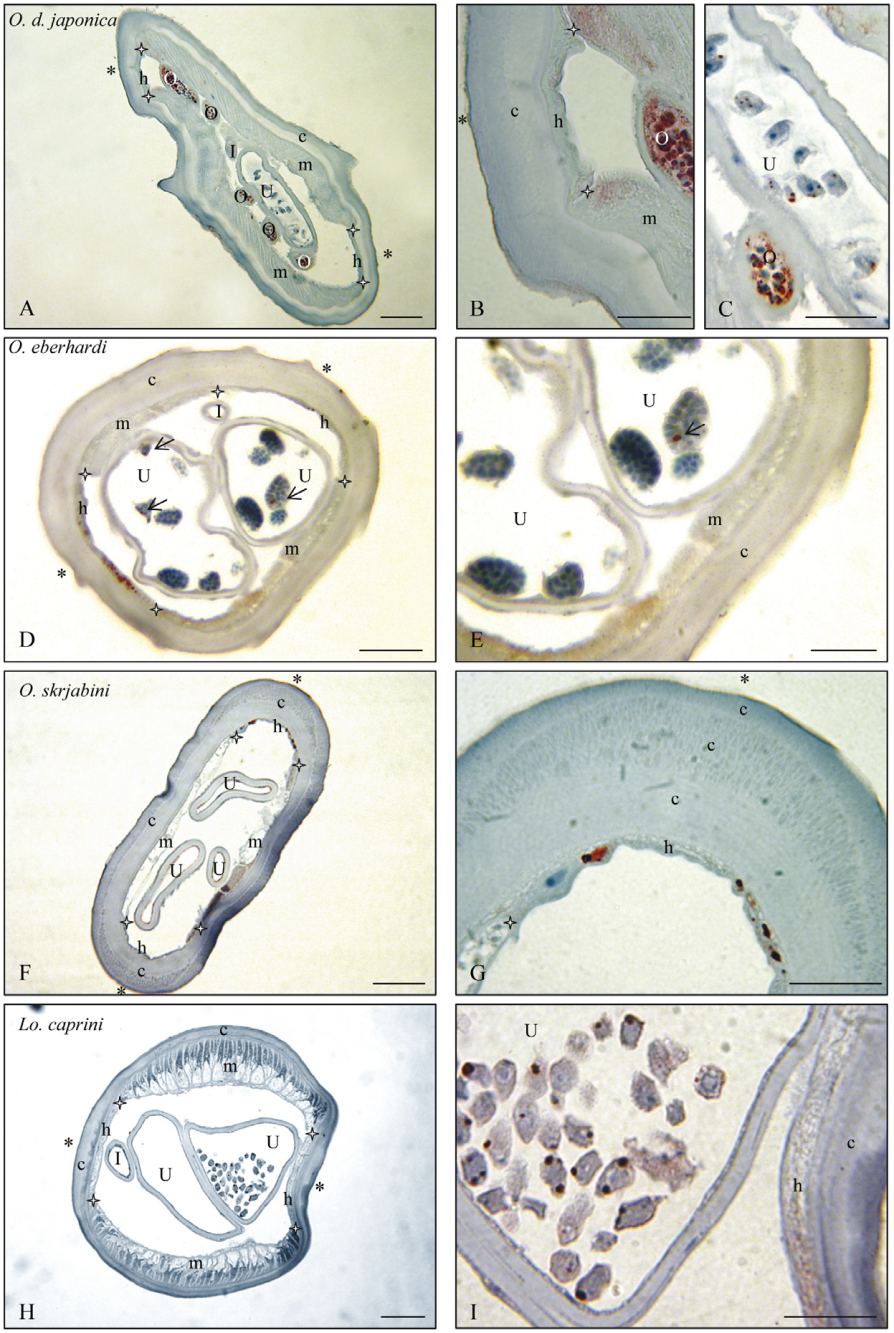
*Wolbachia* immunostaining in the genus *Cercopithifilaria* (C.). Transverse sections of females of four species Wb–, at two magnifications. A. *Cercopithifilaria crassa*: muscles and lateral cuticular crests thick; lateral narrow thick hypodermis and distal ovaries Wb–. B. Detail. C. *Cercopithifilaria longa*: thin lateral chords Wb–, two uteri with microfilariae Wb–. D. Detail. E. *Cercopithifilaria minuta*: thin lateral chords Wb –, thick muscles, two uteri with divided eggs Wb–. F. Detail. G. *Cercopithifilaria shohoi*: lateral crest rather thick, thin hypodermal chord Wb–, small section of ovary with oocytes Wb– and large section of uterus with ova Wb–. H. Detail. I, Intestine; O, Ovary; U, Uterus; c, cuticle; h, hypodermal lateral chords delimitated by white stars; m, muscles; *, lateral plan; white arrow, oocytes. Scale bars: A,C,E,G, 25 µm; B,D,F,H, 10 µm. For staining, see Fig. 1.

**Figure 4 pone-0020843-g004:**
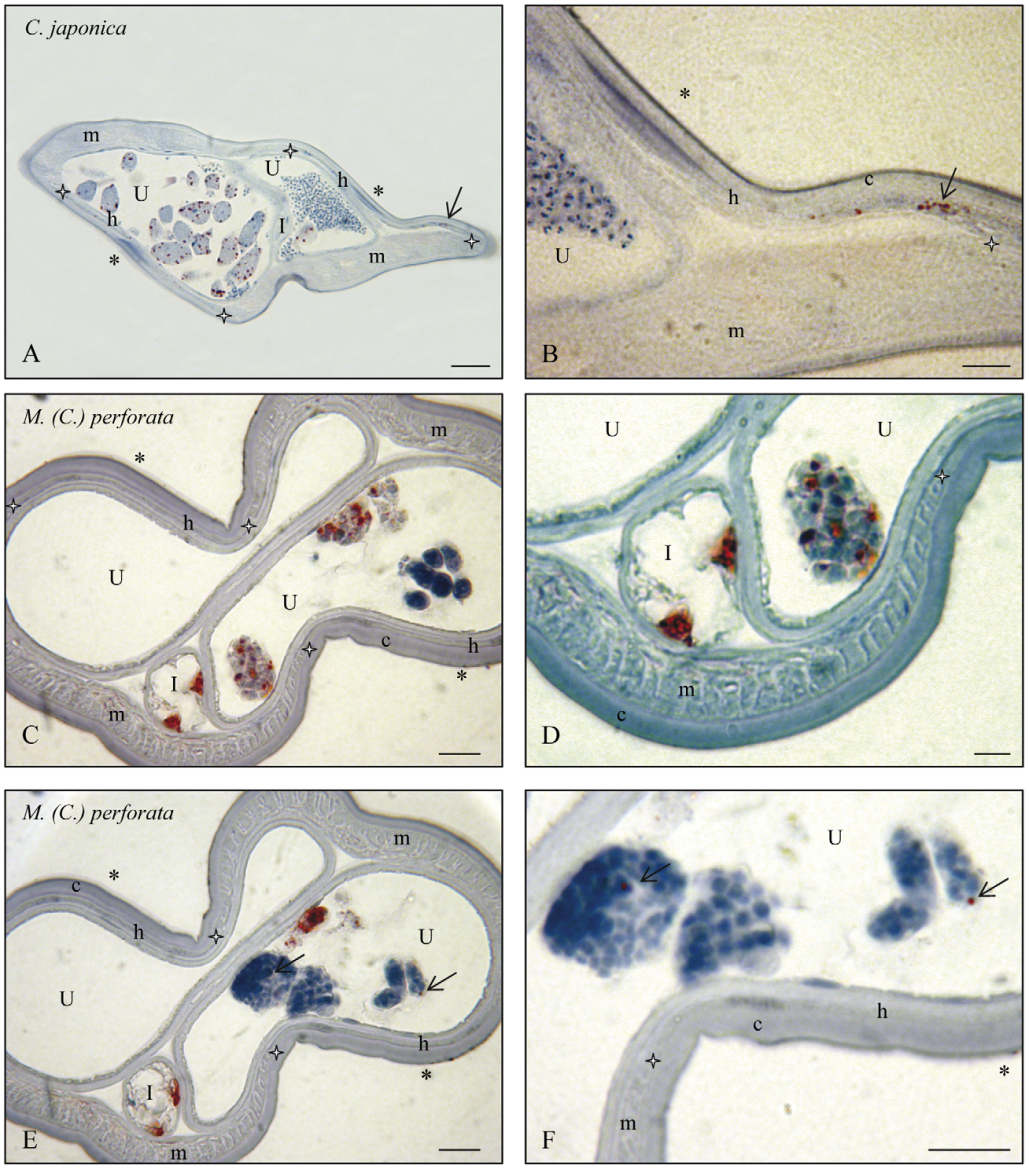
Immunostaining of *Wolbachia* in females of two species. A. *Cercopithifilaria japonica*: wide rather flat lateral crests, hypodermis thin and Wb+, muscles thicker, germ line Wb+. as seen with the intra-uterine ovulae (on left); Wb– spermatozoa in this uterus and in the smaller more distal section (on right). B. Detail. C. *Mansonella (Cutifilaria) perforata*: lateral chords wide, flat and Wb–, muscle cells much thicker, germ line Wb+ as seen with 3 divided eggs in one of the uteri, intestine Wb+. D. Detail. E. Other section, intestine Wb+, eggs Wb+ and hypodermal lateral chord Wb–. F. Detail. I, Intestine; O, Ovary; R, Rachis of ovary; U, Uterus; c, cuticle; h, hypodermal lateral chords delimitated by white stars; m, muscles; *, lateral plan; thin arrow, detail of *Wolbachia*. Scales: A,C,E, bar = 25 µm; B,D,F, bar = 10 µm. For staining, see Fig.1.

**Figure 5 pone-0020843-g005:**
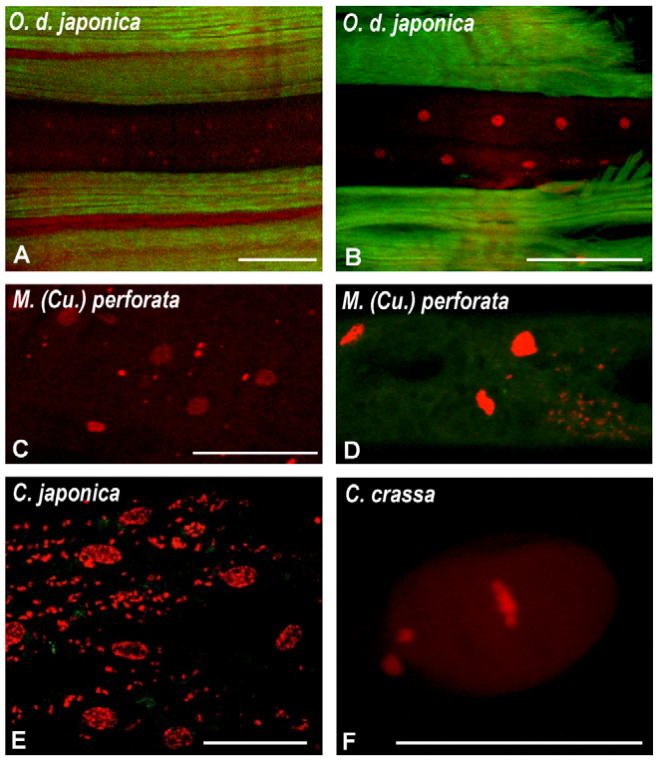
Whole mount fluorescent analysis of *Wolbachia* in females of four species stained with propidium iodide for DNA (in red) and phalloidin for actin (in green). A. Hypodermal lateral chord of a specimen of *Onchocerca dewittei japonica* without *Wolbachia*. B. Idem, a second specimen with a few *Wolbachia* (arrow). C. Epithelial somatic gonad with *Wolbachia* in *Mansonella* (*Cutifilaria*) *perforata*. D. In the same specimen, intestinal wall cells (in) with *Wolbachia* and above, lateral chord (ch) without *Wolbachia*. E. *Wolbachia* in epithelial somatic gonad of *Cercopithifilaria japonica*. F. Metaphase in a *Cercopithifilaria crassa* zygote, showing the absence of *Wolbachia* in the embryo. Polar bodies (PB) on the left. *: host cell nuclei; arrow: *Wolbachia*. Scale bar = 20 µm.

The histoimmunostainings are presented according to the following genera: *Litomosoides* and *Litomosa* (Figure 1), *Onchocerca* and *Loxodontofilaria* (Figure 2), *Cercopithifilaria japonica* and *Mansonella* (Figure 3), other species of *Cercopithifilaria* (Figure 4). Whole mount fluorescent analysis is presented on Figure 5.

The results (Table 1 and 2; Figures 1–7) can be summarised as following:

**Figure 6 pone-0020843-g006:**
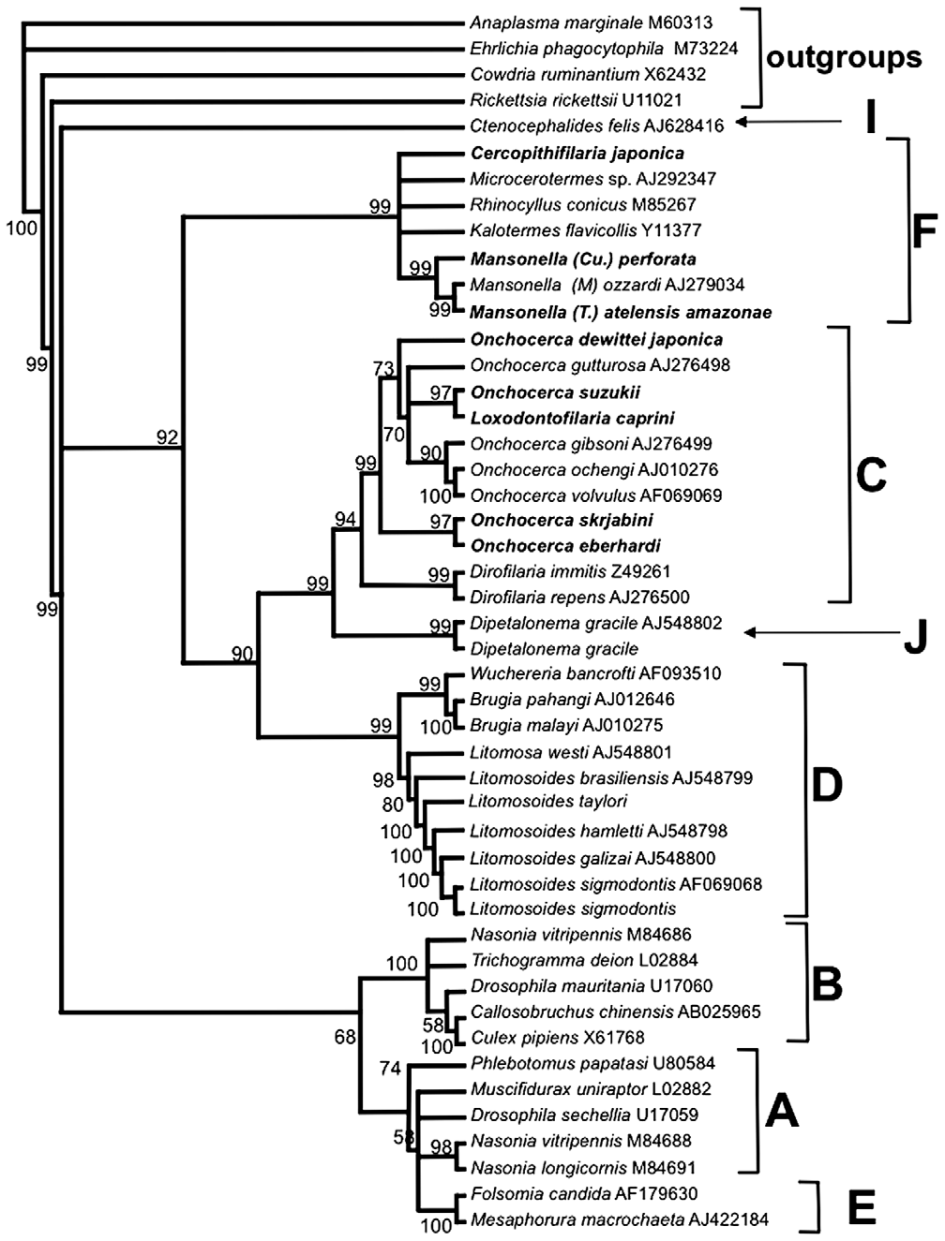
Phylogeny of *Wolbachia* based on 16S rDNA gene sequences. Names at the terminal nodes are those of the host species (with the exceptions of the outgroup species). A–J are the supergroups names according to [1], [3], [8], [14]. The tree has been obtained by Bayesian inference of phylogeny, using MrBayes 2.01; numbers at the nodes are the posterior probability values. Species in bold are those newly found positive in the screening for *Wolbachia* performed in this study. Accession numbers are given for the sequences present in the databases.

**Figure 7:**
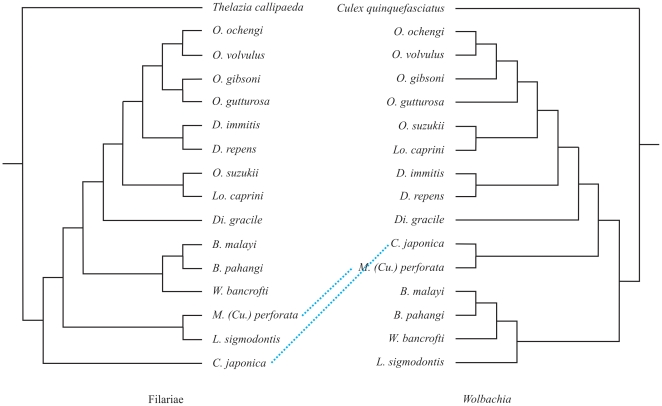
Cocladogenesis of *Wolbachia* and filarial nematodes based on representatives of the species studied. For these species sequences for nematode *coxI* and 12S rDNA (phylogeny on the left) and bacterial 16S rDNA (phylogeny on the right) were available. Dotted lines have been added to emphasize major discrepancies between filarial and *Wolbachia* trees. For further details, see paragraph 7 in the Results section.

**Table 2:** Synthetic results of the screening for *Wolbachia* with PCR, immuno-histo staining and whole mount fluorescent analysis.

					immunostaining/whole mount	
N°	Subfamily	Species	Wb +/n	*Wb* supergroup	Lat. hyp.	Other tissue Wb+
1	**Oswaldofilariinae**	***Piratuba scaffi***	0/3	absent		
2	Waltonellinae	***Ochoterenella royi***	0/4	absent		
3		***Ochoterenella*** ** sp. 1**	0/2	absent		
4	Setariinae	*Setaria digitata*	0/1	absent		
5		***S. tundra***	0/1	absent		
6		**sp. 1**	0/1	absent		
7		**sp. 2**	0/2	absent		
8		**sp. 3**	0/1	absent		
9	Dirofilariinae	***Foleyella candezei***	0/1	absent		
10		*Dirofilaria repens*	1/1	C		
11		*Loa loa*	0/2	absent		
12	Onchocercinae	***Cercopithifilaria bulboidea***	0/2	absent		
13		***C. crassa***	0/4	absent	**−**	
15		***C. japonica***	7/7	F	**+**	som. gonad
14		***C. longa***	0/5	absent	**−**	
16		***C. minuta***	0/3	absent	**−**	
17		***C. multicauda***	0/1	absent		
18		***C. roussilhoni***	0/2	absent		
19		***C. shohoi***	0/4	absent	**−**	
20		***C. tumidicervicata***	0/1	absent		
21		*Dipetalonema gracile*	1/1	J		
22		*Litomosa chiropterorum* *	0/9	Absent	**−**	
23		*Litomosoides sigmodontis*	2/2	D	**+**	
			1/1	D		
24		***L. taylori***	1/1	D		
25		*L. yutajensis*	0/3	absent	**−**	
26		***Loxodontofilaria caprini***	5/9	C	**−**	
27		*M. * ***(Cu.) perforata***	3/4	F	**−**	som. gonad, gut
28		*M. * ***(T.) atelensis amazonae***	1/1	F		
29		***Monanema martini***	0/8	absent		
30		*Onchocerca * ***d. japonica***	4/8	C	**− +**	
31		***O. eberhardi***	4/4	C	**+**	
32		***O. skrjabini***	3/3	C	**+**	
			3/3	C		
33		***O. suzukii***	3/4	C		
34		*O. volvulus*	1/1	C		
35	**Splendidodilariinae**	***Aproctella*** ** sp. 1**	0/1	absent		
			0/1	absent		
	nb of Wb+ specimens/nb of specimens		40/112			
	nb of Wb+ species/nb of species		15/35			

N°: number attributed to species in this study; n = number of specimens screened; Lat. hyp.: lateral hypodermal chords, som.:somatic; Bold characters: species, genus and subgenus newly screened; * Species screened in Junker et al., 2009.

**1. The presence or absence of** ***Wolbachia*** **were confirmed in species previously screened (** **Tables 2** **and S2).** Species previously studied and as expected confirmed positive are: *Onchocerca volvulus* (female worm from a human nodule, Cameroon), *Dirofilaria repens* (from an Italian patient), *Litomosoides sigmodontis* (2 females recovered from a wild *Sigmodon hispidus*, Venezuela) and a less commonly studied species, *Dipetalonema gracile* (a female recovered from *Cebus olivaceus*, collected in Yutaje, Venezuela, like in [5] (Tables 2 and S2). The species confirmed negative were *Loa loa* (two batches of infective larvae recovered from *Chrysops* vectors, Cameroon) and the single *Litomosoides* species that did not harbour *Wolbachia*, *L. yutajensis* (3 females, from the same species, *Pteronotus parnellii,* and locality than in [5]; Figures 1E,F).

**2. Eight newly screened filarial species harbored** ***Wolbachia*** **but, for some of them, not all specimens are positive (** **Tables 2** **and S2).** The species in which *Wolbachia* was detected are the following: *Onchocerca dewittei japonica,* from *Sus scrofa leucomystax* (Figures 2A–C); *O. eberhardi*, from *Cervus nippon* (Figures 2D,E); *O. skrjabini,* from *Cervus nippon* (Figures 2F,G) and *Capricornis crispus*; *O. suzukii* from *Capricornis crispus; Loxodontofilaria caprini,* from *Capricornis crispus* (Figures 2H,I); *Cercopithifilaria japonica*, from *Ursus thibetanus* (Figures 3A,B); *Mansonella (Cutifilaria) perforata,* from *Cervus nippon* (Figures 3C–F); *M. (Tetrapetalonema) atelensis amazonae*, from *Cebus olivaceus* (Tables 2 and S2).

However, in four infected filarial species, *Wolbachia* were not detected in each specimen. These species were *Lo. caprini*, *M. (Cu.) perforata*, *O. d. japonica* and *O. suzukii*. The prevalence of *Wolbachia* varied from 50% to 66% in these species (9, 4, 8 and 4 samples, respectively, see Tables 2 and S2).

**3. In** ***Wolbachia*** **positive filarial species lateral hypodermal chords might be infected, might not be infected, or weakly infected.** The presence of the bacteria in the female germline was not constantly associated to their presence in the lateral hypodermal chords. Among the *Wolbachia* positive species in which the tissue distribution of *Wolbachia* was studied, the lateral chords harboured *Wolbachia* in *L. sigmodontis* (Figures 1A–D), *O. eberhardi* (Figures 2D,E) and *O. skrjabini* (Figures 2F,G), *C. japonica* (Figures 3A,B). On the contrary, *Wolbachia* were not observed in the lateral chords of *Lo. caprini* (Figures 2H,I) and *M. (Cu.) perforata* (Figures 3E,F). In *O. d. japonica*, the bacteria were not detected in lateral chords of sectioned worms (Figures 2A–C) and in one of the two whole mounted worms (Figure 5A), but a few bacteria were observed in the second specimen (Figure 5B).

**4. Rare or novel tissue** ***Wolbachia*** **localizations were observed in** ***Mansonella (Cutifilaria) perforata*** **and** ***Cercopithifilaria japonica*** **.** In *M. (Cu.) perforata* the somatic gonad was found positive once on sectioned worms and this was confirmed on a whole mounted worm; the epithelium lining the gonad harboured the bacteria, but not the external muscle layer (Figure 5C). Moreover, bacteria were constantly found in the cells of the intestine wall, in both sectioned and whole mount material (Figures 3C–F; 5D). *Wolbachia* were also detected in the somatic gonad of *C. japonica*, in whole mounted worms, but not in sectioned worms (Figure 5E); in this species, *Wolbachia* were not found in the intestinal cells.

**5. Four types of** ***Wolbachia*** **were identified in the whole study.** Interestingly, no events of recombination were found in 16S rDNA, *dnaA*, *ftsZ* and *groEL* genes of *Wolbachia* from the sequences of the filarial nematodes studied. According to the *Wolbachia* supergroups, four types of *Wolbachia* were identified in this study: bacteria harboured by *Onchocerca*, *Dirofilaria* and *Loxodontofilaria* species were assigned to supergroup C; bacteria harboured by *L. sigmodontis* and *Litomosoides taylori* were assigned to supergroup D; bacteria harboured by the species of the two subgenera of *Mansonella* (*Tetrapetalonema* and *Cutifilaria*) studied, as well as those harboured by *Cercopithifilaria japonica* were assigned to supergroup F; bacteria harboured by *Dipetalonema gracile* were assigned to supergroup J (Figure 6).

The main *Wolbachia* clusters shown in the 16S rDNA phylogenetic tree of figure 6 were ((*Litomosa*+*Litomosoides*) + (*Wuchereria*+*Brugia*)); (*Dirofilaria*+ (*Onchocerca*+*Loxodontofilaria*)); (*Cercopithifilaria*+*Mansonella+Microcerotermes+Rhinocyllus+Kalotermes + Microcerotermes*). *Wolbachia* from representatives of supergroups A, B and E formed separated and supported clusters; *Wolbachia* from *Ctenocephalides* and *Dipetalonema* formed two separate lineages, that were recently assigned to supergroups I and J respectively [14].

**6.** ***Wolbachia*** **was not detected in more than half of the newly screened species (** **Tables 2** **and S2).** The *Wolbachia* negative species are: in Oswaldofilariinae, *Piratuba scaffi*; in Waltonellinae, *Ochoterenella royi* and *Ochoterenella* sp.1; in Setariinae, the five *Setaria* spp.; in Onchocercinae, 8 (upon nine) *Cercopithifilaria* species (Figures 4A–H, 5F), *Litomosa chiropterorum* (Figures 1G,H), and *Monanema martini*; in Splendidofilariinae, *Aproctella* sp. 1.

**7. Inconsistency between phylogenies of Wolbachia and filarial hosts was evidenced in the cocladogenetic analysis.** The topologies of the trees of the infected filariae and their respective Wolbachia were found to have a significant level of similarity for all of the four tree metrics tested in Component, based on comparisons of the Wolbachia tree with 1000 random host trees (p<0.03 in all cases). The lnL scores of the filarial and Wolbachia ML trees based on the filarial dataset were −6189.35 and −6207.14 respectively. A Shimodaira-Hasegawa test showed that the Wolbachia topology was significantly less likely (p = 0.035) than the filaria topology. The lnL scores of the Wolbachia and filarial ML trees based on the Wolbachia dataset were −10757.38 and −10950.85 respectively. A Shimodaira-Hasegawa test showed that the filaria tree was significantly less likely than the Wolbachia tree (p<0.001). Thus the null hypothesis that the host and parasite have strictly co-speciated was rejected. The major inconsistency was due to the newly screened Cercopithifilaria japonica and Mansonella (Cutifilaria) perforata (Figure 7).

Another point of inconsistency in the phylogenies of hosts and symbionts regards the positioning of L. sigmodontis: while Wolbachia from this filarial is placed as the sister group of endosymbionts from lymphatic filariae (B. malayi, B. pahangi and W. bancrofti), the filaria itself is placed as a deeper branch in the filarial tree (Figure 7). Indeed, based on morphological adult and larval criteria, Litomosoides is closer to lymphatic filariae than to Mansonella [45].

## Discussion

As expected, the screening of a broader and more diversified set of species samples, by a combination of PCR and gene sequencing, immunohistological staining and whole mount fluorescent analysis, revealed new information about *Wolbachia* biology and evolution: novel tissue localizations, strong evidence for recent transfers between unrelated filarial species, and a larger number of species that do not harbour *Wolbachia*. In addition, the occurrence of *Wolbachia* in some members of a species and its absence in others raises questions about the evolution of its obligate requirement [51].

### 1. New filarial tissues infected with *Wolbachia*

It is clear that tissues other than the female germline and the hypodermal lateral chords may be infected with *Wolbachia*. One of these infected tissues is the somatic gonad (epithelial layer), once briefly reported previously [52]. This *Wolbachia* localization was evidenced in *Mansonella (Cutifilaria) perforata* and *Cercopithifilaria japonica* (Table 2; Figures 5C,E). Interestingly, they are both members of the supergroup F of *Wolbachia.*

The real novelty is the *Wolbachia* tissue localization in the intestinal wall; it was only observed in *M. (Cu.) perforata,* but in all sectioned and whole mount samples (Figures 3C–F, 5D).

These divergent localizations suggest a more complex and diversified relationship between the bacteria and filariae. They also raise the question of how and when *Wolbachia* bacteria reach the appropriate filarial host tissues. It is likely that it is an early event, since it was shown in *Brugia malayi* an asymmetric distribution of bacteria in the egg followed by a preferential segregation in defined blastomeres [21].

### 2. Recent capture of *Wolbachia* type F suggested by *Cercopithifilaria japonica*, parasite of the Japanese bear

The species screened in this study generally confirm the previously identified types of *Wolbachia* in the Onchocercidae (Figure 6; see for instance [9]). The newly screened *Loxodontofilaria* is placed among the species of *Onchocerca* [53]. In addition, endosymbionts from this filaria belong to *Wolbachia* supergroup C (Figure 6). There is a major phylogenetic congruence discrepancy between *Wolbachia* and their hosts and it occurs in the genus *Cercopithifilaria* and the supergroup F of *Wolbachia*. One African and seven Japanese species of *Cercopithifilaria* have no *Wolbachia,* while one species in Japan is *Wolbachia* positive. The filarial hosts belong to a well-supported genus, *Cercopithifilaria* as evidenced by adult morphology [45], [54], larval morphology [55], 12S rDNA gene sequences [53], [56], the transmission by hard ticks and the skin-dwelling microfilariae [45]. A parsimonious interpretation of the *Wolbachia* screening is that a single acquisition event took place in *C. japonica.* This hypothesis is supported by the co-cladogenetic analyses (Figure 7).

The supergroup F is intriguing as it is presently the only *Wolbachia* type infecting both insects and onchocercid nematodes [7]. The *Wolbachia* supergroup F contains the species of *Mansonella* studied so far: *M. (M.) ozzardi* and *M. (Esslingeria) perstans* [13], [15], and in this study, *M. (Tetrapetalonema) atelensis amazonae, M. (Cutifilaria) perforata*. *Cercopithifilaria japonica* in supergroup F suggests a transversal transmission event, likely recent due to limited occurrence among the species of this genus. *C. japonica* is a parasite of the Japanese bear in which it coexists with a species of *Mansonella* of the subgenus *Mansonella* [57]. This *Mansonella* species, *M. (M.) akitensis* [58], has not been screened for *Wolbachia* but it likely harbours the type F *Wolbachia*. The bacterial host switching might have occurred between the two filarial parasites of the bear, perhaps via an oral infection route. Indeed filariae, despite their apparent small mouth, can ingest particles from their environment, such as red blood cells [59] and larger bodies, such as microfilariae released in the coelomic cavities of the filarial host [60].

### 3. Absence of *Wolbachia*

The filarial species in which *Wolbachia* were not detected appeared more numerous than it was thought, based upon previous observations. In [34], the percentage of negative species was 10.5% (2 negative among 19), in [5] with a larger sample, it was 37%. In this study, it is twice more elevated, 63%. It has to be emphasized that the negative results were not due to DNA degradations or bad extractions because all of the *Wolbachia* PCR negative samples gave positive amplifications using filarial nematode specific primers. However, we must take into account the fact that, in a few species, *Wolbachia* were not detected in all the specimens. This can partly be explained in species which do not harbour *Wolbachia* in the lateral chords, or at very low density, as *Onchocerca dewittei japonica* (Figures 5A,B) and *Loxodontofilaria caprini* (Figures 2H,I). In *M. (Cu.) perforata,* the bacteria are in the intestine wall of the female worms (Figures 3C,D, 5D) and any part of female body would be *Wolbachia* positive, but this is not the case (Tables 2, S1 and S2). Thus, in *M. (Cu.) perforata*, presence/absence of *Wolbachia* may occur, as suggested by [15] for another *Mansonella* species, *M. (Esslingeria) perstans* from humans. It is interesting to note that in both of these cases, the *Wolbachia* supergroup is F. Further, research performed on deeply studied filariae, such as *Brugia malayi*, have also shown that the amount of *Wolbachia* carried by a worm may vary greatly over time and be stage-dependent [21]. This dynamic probably has little impact when considering developing larval stages, because these are transient and the chance of recovering them in the wild is extremely low. More interesting is the observation that the female worms recovered in the wild are not all fully gravid in some species (Figure 2F). This was not the case of filariae from frogs, lizards, bats, birds, but was the case from some parasites of Japanese ungulates. It is worth to note that we paid great attention to the part of worm sampled to ensure that the germline was screened in all species.

### 4. Systematic position of *Wolbachia* negative filariae

It is clear that there is a need to increase the number of screened specimens for more solid results. However, in several cases, the global results are impressive and the distribution of the *Wolbachia* negative species does not appear random. As a matter of fact, at present, the species parasitic in frogs and lizards are negative for *Wolbachia* if they are Waltonellinae (three species of *Ochoterenella*), Oswaldofilariinae (one species of *Piratuba*), or Dirofilariinae (two species of *Foleyella*). It was also shown here that the first screened species parasitic in birds, an *Aproctella* in the Splendidofilariinae, was *Wolbachia* negative (7 females screened with PCR, from two passeriform species, totalising 7 specimens hosts). *Wolbachia* were not detected also in several species of Onchocercinae from mammals. The first is *Litomosa chiropterorum,* from an African bat (8 specimens screened from 8 *Miniopterus schreibersi*), an unexpected observation since the single *Litomosa* species screened previously, *Li. westi* from a North American rodent Geomyoidea, was *Wolbachia* positive [5].

The second species is *Monanema martini* (8 females screened from 8 murid specimens). The absence of the bacteria is an important feature, considering that this filaria was used, in the past, as a model of onchocerciasis, a true limit due to the important role played by the bacteria in the pathology of the disease [39], [61]–[63]. The absence of *Wolbachia* could explain partly the weak ocular pathology induced in the murid host [49]. Severe or mild ocular onchocerciases have been related to a strain-dependent variation of density of *Wolbachia* per filarial genome [64].

Other species in which *Wolbachia* were not detected belong to the genus *Cercopithifilaria*; 8 out of 9 species were *Wolbachia*-negative (Table 2; Figures 4; 5F).

Two hypotheses might be taken into account to explain the absence of *Wolbachia*. In the first case, *Wolbachia* could be considered to be present and then were subsequently lost [38]. This could have occurred before the mutually dependent symbiosis between symbiont and host developed. The second hypothesis is that the bacteria were not yet acquired within that filarial group.

The first hypothesis seems to possibly explain some of the observed cases, such as *Litomosoides yutajensis* (5 samples: 2 males previously and now 3 more females), a species without *Wolbachia*, in contrast with 5 congeneric species infected with *Wolbachia*. In the cases of *Acantocheilonema viteae* and *Onchocerca flexuosa* [38], the absence of *Wolbachia* appears to be a secondary loss, because some genes of the bacteria were incorporated in the filarial genomes. However, it is not clear whether this loss occurred before symbiosis was established or not. This is of interest because if loss was after symbiotic establishment, perhaps some of the genes incorporated into the host genome were those required by the host now provided by *Wolbachia*, but the extent of this event is still to be understood.

The hypothesis that the *Wolbachia* negative species might never have been infected, might be considered for the filarial groups that are supposed to be “ancient” such as the Oswaldofilariinae [44], [45], [65]. Estimation of dates of divergence has been proposed for some groups of nematodes based on molecular phylogenetic analyses [66]. In filariae, data from morphology, biology, geographic distribution, host range and palaeontology led to the proposal that the Oswaldofilariinae emerged during the late Jurassic, at the beginning of the Gondwanian break up, 140 mya [44], [45]. This is before the hypothesized ancestral acquisition of a *Wolbachia* by an onchocercid [3]; it follows that the absence of *Wolbachia* in Oswaldofilariinae could be primitive.

*Foleyella* is another parasite of saurians, which appears to have no *Wolbachia*; it is presently placed in the Dirofilariinae, which includes the *Wolbachia* positive *Dirofilaria*, but this systematic position needs to be revised because the characters of the infective larvae are very distinct [55]. A solid phylogeny of Onchocercidae linking traditional and molecular data is needed and warrants further investigations.

The subfamily Setariinae, parasitic in ungulates and in which *Wolbachia* were not detected, as first shown by [67], also deserves a comment. Based on larval morphology, it has been hypothesized that it evolved separately from the other onchocercids and derived from a group of spirurid Habronematinae [65]. Until now no spirurids have been found infected with *Wolbachia* [5], [34].

Did the bacterial infection never occur, or was the useful part of *Wolbachia* genome incorporated in the host genome and subsequently the bacteria eliminated, to reply to some adverse constraint? Further genomic analyses will solve this question concerning the absence of *Wolbachia* in ancestral filariae.

At present, the features observed on tissue or specimen distribution, and a very probable recent lateral transfer, suggest complex evolutionary dynamics of interactions between the symbionts, their host filariae and the nematode hosts. In the sampling studied, which will be further enlarged, it may become possible to use some defined species to decipher these questions.

## Materials and Methods

### Specimens and species

Specimens of filariae were recovered during dissections of the vertebrate hosts captured in the wild from different geographic areas [50], [54], [68]–[72].

All experiments, procedures and ethical issues were conformed to the competent national ethical bodies: Venezuelian animals were captured according to Licencia con Fines Cientificos N° 2192 dated June 18, 2007 and Contrato Marco Acceso Recursos Genéticos N° 33/2007 both granted by Ministerio del Ambiente de la Republica Bolivariana de Venezuela. Japanese serows, sika deer, bears, and wild boars were killed by hunters who have an individual permit to kill wild animals in accordance with the conservation and control policies of the Ministry of the Environment of Japan. Italian samples were collected by veterinarians and physicians and no permits were necessary. African rodents and agama do not belong to protected species and were obtained from local hunters. African mountain reedbuck, plain zebra, gemsbok and porcupine were also obtained from local hunters. Bats from South African bats have been collected for previous studies [48] in which no permits were necessary. Brazilian birds were donated by the Brazilian Institute of the Environment and Renewable Resources (IBAMA), Region of Juiz de Fora, Minas Gerais for the laboratory of Taxonomia and Ecology of Helminths of the Department of the Zoology.

Many of the filariae from large mammals were extracted from the subconnective tissue, dermis, or tendons of limbs. Host animals kept at 4°C were sent to the laboratory after they were killed. Afterwards the parts of the animals were dissected to collect living filarioids for *Wolbachia* study. From frogs, lizards, birds, bats and rodents filariae were generally recovered immediately after host death. Samples used for positive and negative PCR controls were laboratory strains.

Species identification of the specimens was done with morphological studies performed by several of us (OB, SU, RG, SL, MD). Some species are not yet named, but all are under study, and morphological analysis and sequencing of *coxI* gene in this study and in [53] showed that they represent distinct molecular entities. Co-infection of a host specimen by several congeneric or non-congeneric filarial species was rather frequent in large mammals, but in a few cases, the same filarial species was recovered from two host species. The supraspecific levels of taxonomy followed the systematic works of [46], [73],[74] and more recent studies for some taxa: [65], [71], [75] for the subgeneric divisions of *Mansonella* Faust, 1929; [54], [56] for the genus *Cercopithifilaria* Eberhard, 1980 (created as a subgenus); and [54] for the genus *Loxodontofilaria* Berghe & Gillain, 1939.

Abbreviations used in the text are: *A.* for *Aproctella*; *C.* for *Cercopithifilaria*; *Cu*. for *Cutifilaria*; *D.* for *Dirofilaria*; *Di.* for *Dipetalonema*; *F*. for *Foleyella*; *L*. for *Litomosoides*; *Li*. for *Litomosa*; *Lo*. for *Loxondontofilaria*; *M*. for *Mansonella*; *Mo*. for *Monanema*; *O*. for *Onchocerca*; *Oc*. for *Ochoterenella*; *P*. for *Piratuba*; *S*. for *Setaria*; T. for *Tetrapetalonema*. The material screened in this study is detailed in Figure S1, Tables S1 and S2.

Filarial worms were fixed and kept in absolute alcohol at 4°C for PCR analyses, in 4% PFA (paraformaldehyde) for overnight at 4°C for immunohistochemical staining and whole mount fluorescent analysis. In many cases, filarial specimens were cut into anterior (a), median (m) and posterior (p) parts, which were fixed for the different analysis approaches. Since *Wolbachia* are transmitted by female filariae, almost all of the studies were based on female worms; male specimens were rarely examined, and we analysed this sex alone in a single case, *Di. gracile* (Table S3).

### Molecular screening for *Wolbachia* on filarial nematodes

PCR screening for *Wolbachia* was conducted according to [5], [12], using general *Wolbachia* primers for 16S rDNA (99f and 994r [76]), originally designed to work on *Wolbachia* from the supergroups A and B, and primers for 16S rDNA (16SWolbF and 16SWolbR3), originally designed to work on *Wolbachia* from the supergroups A–D [12], but whose target sites are also conserved in *Wolbachia* from supergroups E and F [8].

PCRs were performed in a 20 µl final volume under the following conditions: 1x buffer (containing 1.5 mM MgCl_2_, Eppendorf™), 0.2 mM of each dNTP, 1 µM of each primer, and 0.5 U of Taq DNA Polymerase (Eppendorf™). The thermal profile used was: 94°C 45 sec, 52°C 45 sec, and 72°C 90 sec for 40 cycles.

When the PCRs were negative under the above PCR conditions, a nested-PCR approach was implemented in order to improve the sensitivity of the PCR screening [5], [77]. The first PCR was performed using the general eubacterial primer 27F [78] combined with 16SWolbR3; PCR volumes and conditions were as above. Five µl were visualised on a 1.5% w/v agarose gel and one µl of the first PCR was diluted 1/10 and 1/100 in water, and then both used as templates in a second PCR, performed using primers W-EF and W-ER [70]. W-ER and W-EF recognize sites that are conserved in supergroups E–F and that are internal to the primers used in the first PCR. PCR conditions for this amplification were as described in [79].

Of samples remaining negative after the two PCRs approaches described above, PCRs with primers 16SWolbF and 16SWolbR3 were performed varying the following parameters: MgCl_2_ concentrations at 2.5, 4 and 6 mM and annealing temperatures of 52°C +/−5°C.

DNA preparations from filarial species harbouring *Wolbachia* (*D. immitis* and *Brugia pahangi*) and from a *Wolbachia*-infected strain of mosquitoes (*Culex pipiens*) [5] were included in the screening as positive controls. DNA preparations from a filarial species not harbouring *Wolbachia* (*A. viteae*) [5] were included as negative controls.

Of the samples positive for PCR screening homologous to *Wolbachia* 16S rDNA, *dnaA*, *ftsZ* and *groEL* were also amplified using the primers described in [5], [80] under the following conditions: 1x Eppendorf buffer including 1.5 mM MgCl2, 0.2 µM of each dNTP, 1 µM each of forward and reverse primers, and 0.5 units MasterTaq (Eppendorf). The thermal profiles we used were: (1) *dnaA*, 94°C 45 sec, 52°C 45 sec, and 72°C 90 sec, for 40 cycles; (2) *groEL*, 94°C 45 sec, 60°C 45 sec, 72°C 80 sec, for 5 cycles, and 94°C 45 sec, 55°C 45 sec, and 72°C 80 sec, for 34 cycles; (3) *ftsZ*, 94°C 30 sec, 60°C 45 sec, 72°C 90 sec, for 5 cycles, and 94°C 30 sec, 57°C 45 sec, and 72°C 90 sec, for 34 cycles.

Amplifications were performed in 20–50 µl volumes.

In all cases, in order to ascertain the DNA conditions before *Wolbachia* screenings and to confirm morphological identification, *coxI* amplification was performed as described in [12].

### Sequencing conditions

*coxI* sequencing was performed as described in [12]. *dnaA, ftsZ* and *groEL* sequencing were performed as described in [5], [80]. From the *Wolbachia* PCR positive samples, almost the full length of the 16S rDNA gene of *Wolbachia* was sequenced using primers 27F and 16SWolbR3. The amplifications obtained (about 1400 bp) were gel-purified (using the QIAquick® PCR Purification Kit, Qiagen) and directly sequenced using ABI technology. The sequences obtained have been deposited in the EMBL Data Library. The *dnaA*, *groEL* and *ftsZ* sequences were not obtained from all the taxa included in this study mainly caused by the scarcity of certain specimens, and amplification/sequencing problems for some of the species examined. Where DNA of the host was amplified, the PCR product was purified as above and directly sequenced using ABI technology.

A list of the sequences including accession numbers is available in Table S2.

### Immunohistochemical staining of worm sections

Immunohistochemical staining was performed according to [24], [48]. A rabbit polyclonal antiserum raised against the WSP of the *Wolbachia* from *B. pahangi* (designed by [81]) has been used to stain samples of 13 filarial species (see Tables S2 and S3). After paraffin inclusion, 4 µm sections were obtained and placed on Silane coated glass slides (3-aminpropyltriethoxysilane) and then kept at 63°C overnight, to avoid sections detaching from slides. To verify that the 63°C overnight did not alter the specific epitopes, *Wolbachia* positive *L. sigmodontis* laboratory strain were incubated at both 63°C and room temperature overnight and then stained: no significant differences in the staining were observed. Negative controls were carried out by omitting the primary antibody.

Fixed female worms were divided in three main parts, posterior (p), median (c) and anterior (a) in order to observe the different regions of the genital tract. Transverse sections were made at different levels of each part, and few of them were stained with hemalun-eosin for anatomical identification. Lateral cuticular internal crests were identified to orient the worm section; hypodermal chords extend above and on the side of the crests. The filarial species used for histology are opisthodelphic and the initial part of the ovaries is in the posterior part of the worm; the distal region of the ovary is composed of a cytoplasmic axis, the rachis [82], [83], and an outer cytoplasmic layer with the nuclei of oogonia and oocytes (in the text we will refer to these states as oocytes, and germline to describe the whole production of the gonad). An epithelial layer and an outer muscle layer surround the gonad, both referred to as somatic gonad in the text [25]. Uteri occupy almost the whole body and are found in median and anterior part of worms. The different uterine contents are ovulae, spermatozoa, divided eggs and microfilariae. Eggs were identified as aborted by hemalun-eosin staining when divided eggs were eosinophilic and nuclei not discernible. The laboratory strain of *Litomosoides sigmodontis,* which has been shown in several studies to harbour *Wolbachia* [84]–[86] was used as a positive reference for *Wolbachia* immunostaining.

### Whole mount fluorescent analysis

Worms were cut with a razor blade to expose the different tissues to RNAse A (15 mg/mL, Sigma) in rotating tubes overnight at 4C. They were rinsed in PBS, and incubated with a fluorochrome-conjugated Phalloidin (atto−488 Phalloidin, Fluka, at 10 nanomolar) overnight to stain F-actin, followed by a Propidium Iodide (Molecular Probes, 1.0 mg/mL solution) incubation for DNA staining for 20 minutes in PBS (1∶50) and a 5 minutes wash. Tissues were mounted in Vectashield (Vector Laboratories) [25]. The species analyzed were *C. crassa*, *C. japonica, Lo. caprini, M. (Cu.) perforata* and *O. d. japonica* (Table S1).

### Phylogenetic reconstruction

The bacterial 16S rDNA, *dnaA*, *groEL* and *ftsZ* sequences and filarial 12S rDNA and *coxI* sequences generated were aligned with the sequences available in the databases (for the ribosomal genes according to their secondary structures) using ClustalX2 [87].

The alignments were analysed using Maximum Likelihood (ML) and Bayesian Inference of phylogeny (BI) methods. The appropriate model of sequence evolution for ML and BI was estimated via likelihood ratio test using Modeltest 3.7 [88]: the model selected for the filarial concatenated dataset was HKY+G, while the model for the *Wolbachia* concatenated dataset was GTR+I+G. Phylogenetic analyses were performed using PAUP* 4.0 b10 [89] and MrBayes [90]. In addition, a phylogenetic tree was inferred with a GTR+Γ_4_ nucleotide substitution model in a Bayesian framework using MrBayes version 3.0 [90]. Two independent runs were performed, each using 1 million steps with four chains sampling every 100 steps. The first 10% of the trees were removed and posterior probabilities were calculated from these post-burnin trees.

### Test for recombination

Four *Wolbachia* alignments (relative to the genes 16S rDNA, *ftsZ*, *groEL* and *dnaA*) were screened for presence of recombination events by using a set of nonparametric detection programs: RDP, GENECONV, Bootscan, MaxChi, Chimaera, SisScan and LARD [91]–[97]. The first six programs search for putative recombination breakpoints in a set of aligned DNA sequences and are implemented in RDP3 software package, whilst LARD checks signals detected by other methods [92]. Sequences were auto-masked for optimal recombination detection. General recombination settings were as follows: sequences were considered linear and the highest acceptable P-Value was set to 0.01 and overlapping signals were disentangled.

Method specific options were as follow: MaxChi and Chimaera were run with a variable window size; Bootscan and SisScan were forced for exploratory screening. Successive phases of refining analysis and manual tests were performed where needed.

### Cocladogenesis analysis

To test for congruence, phylogenies for the hosts (based on a concatenated dataset of *coxI* and 12S rDNA) and for the bacterium (based on a concatenated dataset of 16S rDNA, *ftsZ*, *dnaA* and *groEL*) were compared with two methods. The first tested whether there was a greater than random correspondence between reconstructed nodes for host and symbiont. This was performed in Component (R. Page, University of Glasgow, UK) using the “compare tree with” function, with 1,000 randomized trees and the four available tree-comparison metrics (partition; triplets; quartets, nearest neighbour interchange). The second test examined the null hypothesis that the endosymbionts have undergone cocladogenesis with their hosts. ML trees for each dataset were first estimated using the successive approximation method [89]. Then, the scores for each of these ML trees based on the host dataset were compared using the [98] test. This was repeated using the parasite dataset. A single uninfected filarial (*T. callipaeda*) and a non-filarial *Wolbachia* (from *C. quinquefasciatus*) were used as outgroups.

## Supporting Information

Figure S1 Position of the genera screened in the present study indicated on a schematic representation of a key of the onchocercid subfamilies, based on morphological characters (following [46]). Total number of genera per subfamily listed. *Genus screened for the first time. ** Two subgenera in *Mansonella*.
(DOC)

Table S1 Details of material studied with PCR, immunostaining assays (IHS) and whole mount fluorescent analysis (fluo). The scheme follows the classification as in Tables 1 and 2. Species, genus and subgenus, subfamily are in bold characters when newly screened; specimens ids are in bold characters when female worms; m = male; f = female; a = anterior part; c = central part; p = posterior part; *150 infective larvae; +  = *Wolbachia* positive specimens.
(DOC)

Table S2 Results of *Wolbachia* screening based on PCR, immunostaining assays and whole mount fluorescent analysis in 35 filarial nematodes. Taxa are presented in alphabetical order.
(DOC)

Table S3 *Wolbachia* distribution in the tissues of 13 onchocercid species. +: stained; −: not stained; NA: not available because the body structure is not present. *staining shown on Figures 1–4.
(DOC)
