# Supplementary material for: New Insights into the Evolution of Wolbachia Infections in Filarial Nematodes Inferred from a Large Range of Screened Species
Source: PLoS One. 2011 Jun 22;6(6):e20843. doi: 10.1371/journal.pone.0020843 (PMC3120775; doi:10.1371/journal.pone.0020843)
Supplement: Figure S1 — Position of the genera screened in the present study indicated on a schematic representation of a key of the onchocercid subfamilies, based on morphological characters (following [46]). Total number of genera per subfamily listed. *Genus screened for the first time. ** Two subgenera in Mansonella. (DOC) [file pone.0020843.s001.doc]

**Figure S1**

Position of the genera screened in the present study indicated on a schematic representation of a key of the onchocercid subfamilies, based on morphological characters (following Anderson and Bain, 2009). Total number of genera per subfamily listed.


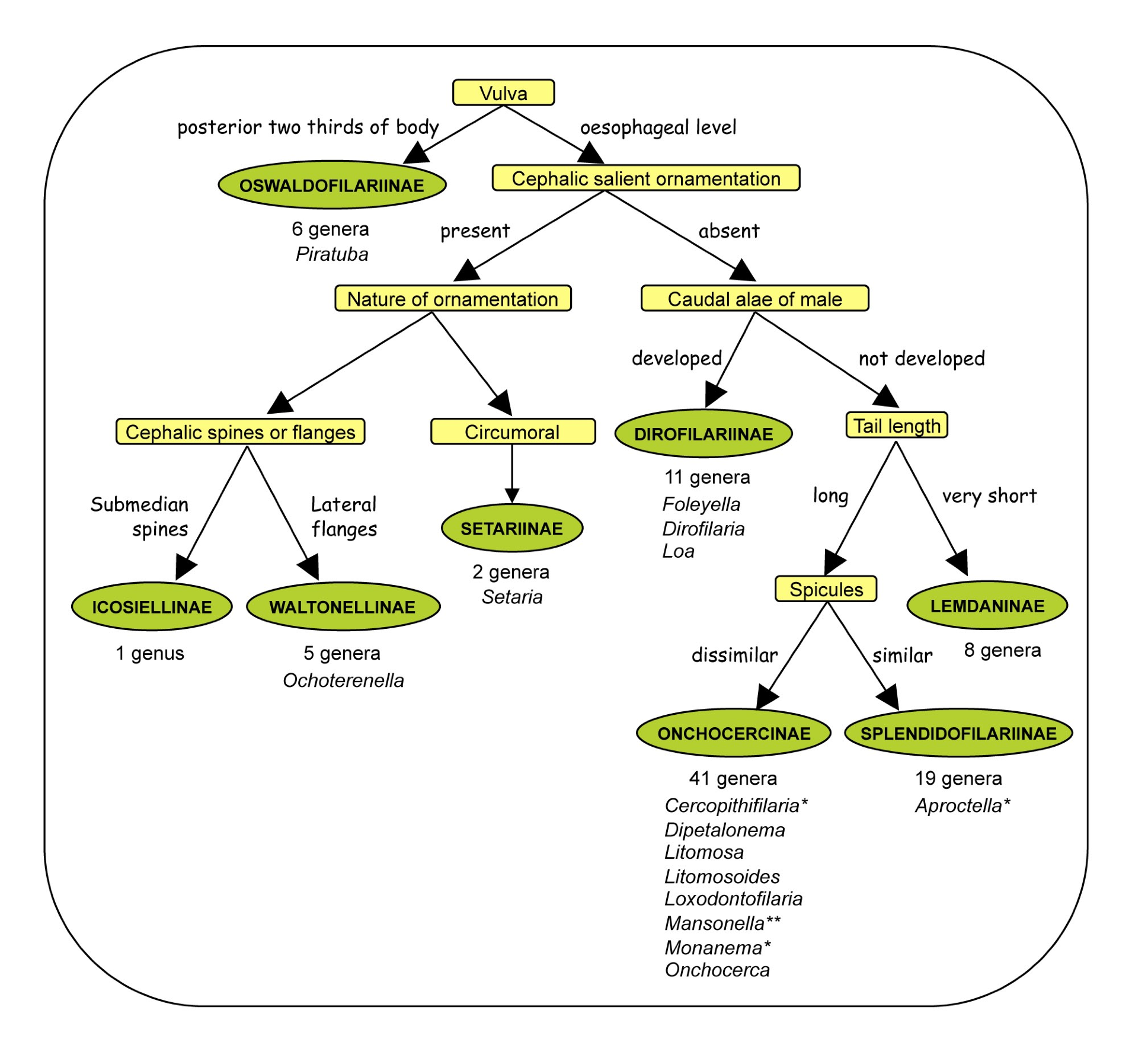


* Genus screened for the first time. ** Two subgenera in *Mansonella*.
