# Supplementary material for: New Insights into the Evolution of Wolbachia Infections in Filarial Nematodes Inferred from a Large Range of Screened Species
Source: PLoS One. 2011 Jun 22;6(6):e20843. doi: 10.1371/journal.pone.0020843 (PMC3120775; doi:10.1371/journal.pone.0020843)
Supplement: Table S1 — Details of material studied with PCR, immunostaining assays (IHS) and whole mount fluorescent analysis (fluo). The scheme follows the classification as in Tables 1 and 2. Species, genus and subgenus, subfamily are in bold characters when newly screened; specimens ids are in bold characters when female worms; m = male; f = female; a = anterior part; c = central part; p = posterior part; *150 infective larvae; + = Wolbachia positive specimens. (DOC) [file pone.0020843.s002.doc]

**Table S1**

Details of material studied by PCRs, immunohistostaining (IHS) or whole mount fluorescent analysis (fluo)

| N° | Subfamily | Species | Specimen Ids - PCRs | Specimen Ids - IHS | Specimen Ids - fluo |
| --- | --- | --- | --- | --- | --- |
| 1 | **Oswaldof.** | ***Piratuba scaffi*** | **34YU-1 (f); 34YU-2 (f)**; 34YU-3 (ma) |  |  |
| 2 | Walton. | ***Ochoterenella royi*** | **10YU-1; 10YU-2** |  |  |
| 3 |  | ***Ochoterenella* sp. 1** | **193JW (f); 194JW (f)** |  |  |
| 4 | Setar. | *Setaria digitata* | **ST1 (f)** |  |  |
| 8 |  | ***S. tundra*** | **SET1** |  |  |
| 5 |  | **sp. 1** | *88YU* |  |  |
| 6 |  | **sp. 2** | 85YU; **86YU** |  |  |
| 7 |  | **sp. 3** | **84YU** |  |  |
| 9 | Dirofil. | ***Foleyella candezei*** | **68CE (f)** |  |  |
| 10 |  | *Dirofilaria repens* | PIS |  |  |
| 11 |  | *Loa loa* | 131JW; *132JW* (poolL3)* |  |  |
| 12 | Onchoc. | ***Cercopithifilaria bulboidea*** | **C1-3 (f); C1-4A (f)** |  |  |
| 13 |  | ***C. crassa*** | **S51-PB6 (f)** | **S61-M5 (fc); S61-M4C (fp)** | **S20-AB24** |
| 14 |  | ***C.japonica*** | **BP5-1 (f)^+^; BS9-1 (f)^+^; BS6-2 (f)^+^** | **BP5-3 (fc)^+^; GB10-N6 (fp)^+^; GB10-3 (fa)(fc)(fp)^+^** | **GB43-B5^+^** |
| 15 |  | ***C.longa*** | **S51-PB1 (f)**; S51-PB2 (m); **AG1-5 (f); AG1-10 (f)** | **S61-M22 (fc)** |  |
| 16 |  | ***C.minuta*** | **C1-A4 (f); SW1-23 (f)** | **C1-A5 (fp)** |  |
| 17 |  | ***C.multicauda*** | **G119(f)** |  |  |
| 18 |  | ***C.roussilhoni*** | **143SE-1 (f)** |  |  |
| 19 |  | ***C.shohoi*** | **SW21-170 (f); SW1-32 (f); C1-LB4 (f);** | **C1-PLL3 (fp)** |  |
| 20 |  | ***C.tumidicervicata*** | **SW1-9 (f); SW5-119 (f); C1-LB8 (fa); C1-LBB1 (f)** |  |  |
| 21 |  | *Dipetalonema gracile* | 15YU (ma) |  |  |
| 22 |  | *Litomosa chiropterorum* | 252JW(m); **253JW(f); 254JW(f); 264JW(f); 267JW(f); 268JW(f); 274JW(f)** | **254JW-1 (fa); 254JW-2 (fc); 254JW-3 (fa)** |  |
| 23 |  | *Litomosoides sigmodontis* |  | **32BX-1 (fc)(fp)^+^; 32BX-2 (fc)(fp)^+^** |  |
|  |  |  | 21YU^+^ |  |  |
| 24 |  | ***taylori*** | 44YU^+^ |  |  |
| 25 |  | *yutajensis* | **39YU (f)** | **102CV-1 (fa)(fp); 102CV-2 (fa)(fc)(fp)** |  |
| 26 |  | ***Loxodontofilaria caprini*** | **C1-1A (fa)^+^; C1-FFL1 (fa); C1-SB10 (f); YG3-12 (f); YG2-25 (f)^+^; YG3-1 (f)^+^** | **C1-1 (fc); C1-A2 (fc)^+^** | **10-9^+^; 101^+^** |
| 27 |  | *M.* ***(Cu.) perforata*** | **S51-PB5 (f)^+^; S51-PB9 (fa)** | **S51-PB8 (fc)(fp)^+^** | **S20-PSA1 ^+^** |
| 28 |  | *M.* ***(T.) atelensis amazonae*** | **15YU (fa)^+^** |  |  |
| 29 |  | ***Monanema martini*** | **324NB; 332NB; 342NB; 350NB** |  |  |
| 30 |  | *Onchocerca* ***d. japonica*** | **B59-1 (fa)^+^; B61-4 (fa); B61-7 (fa)^+^** | **B59-11 (fa); B59-3 (fc); B59-15 (fp) ^+^; B61-10 (fc)** | **B107-1; B114 ^+^** |
| 31 |  | ***O. eberhardi*** | **S51-9** (f)^+^ | **S51-13 (fc)^+^; 1-MO9-F2 (fc)^+^; 2-MO9-F2 (fc)^+^** |  |
| 32 |  | ***O. skrjabini*** | **S51-2 (f)^+^; S51-4 (f)^+^;** S51-7 (ma) | **S51-5 (fc) ^+^** |  |
|  |  |  | **C1-FL5 (f)^+^; SW30-26 (f); G30 (f)** |  |  |
| 33 |  | ***O. suzukii*** | **YG2-35 (f)^+^; YG2-37 (f)^+^; YG2-53 (f)** |  |  |
| 34 |  | *O. volvulus* | **86JW (J1)^+^** |  |  |
| 35 | **Splendid.** | ***Aproctella* sp. 1** | **45YU-1, 45YU-2,116YU, 117YU** |  |  |
|  |  |  | **76YU-1, 76YU-2** |  |  |

The scheme follows the classification as in Tables 1 and 2. Species, genus and subgenus, subfamily are in bold characters when newly screened; specimens ids are in bold characters when female worms; m = male; f = female; a = anterior part; c = central part; p = posterior part; ***150 infective larvae; ^+^ *Wolbachia* positive specimens.
