# Supplementary material for: New Insights into the Evolution of Wolbachia Infections in Filarial Nematodes Inferred from a Large Range of Screened Species
Source: PLoS One. 2011 Jun 22;6(6):e20843. doi: 10.1371/journal.pone.0020843 (PMC3120775; doi:10.1371/journal.pone.0020843)
Supplement: Table S2 — Results of Wolbachia screening based on PCR, immunostaining assays and whole mount fluorescent analysis in 35 filarial nematodes. Taxa are presented in alphabetical order. (DOC) [file pone.0020843.s003.doc]

**Table S2**

Investigated specimens. List of samples including specie name (if available), voucher number, collection ID number and accession numbers of the nematodes and Wolbachia included in this study.

| **Molecular voucher** | **ID Number** | **Species** | **Nematodes sequences** | | **Wolbachia sequences** | | | |
| --- | --- | --- | --- | --- | --- | --- | --- | --- |
|  |  |  | **coxI** | **12SrDNA** | **16SrDNA** | **dnaA** | **ftsZ** | **groEL** |
| MIB:Zpl:01523 | 45 YU-1 | *Aproctella* sp. 1 | FR823333 | FR827902 |  |  |  |  |
| MIB:Zpl:01524 | 45 YU-2 | *Aproctella* sp. 1 | FR823334 | FR827903 |  |  |  |  |
| MIB:Zpl:02719 | 116 YU | *Aproctella* sp. 1 | FR823331 | FR827904 |  |  |  |  |
| MIB:Zpl:02720 | 117 YU | *Aproctella* sp. 1 | FR823335 | FR827905 |  |  |  |  |
| MIB:Zpl:01521 | 76 YU-1 | *Aproctella* sp. 1 |  | FR827900 |  |  |  |  |
| MIB:Zpl:01522 | 76 YU-2 | *Aproctella* sp. 1 | FR823332 | FR827901 |  |  |  |  |
| MIB:Zpl:00940 | C1-3 | *Cercopithifilaria bulboidea* | AM749247 | AM779779 |  |  |  |  |
| MIB:Zpl:00942 | C1-4a | *C. bulboidea* | AM749248 | AM779780 |  |  |  |  |
| MIB:Zpl:00925 | S51-PB6 | *Cercopithifilaria crassa* | AM749260 | AM779791 |  |  |  |  |
| MIB:Zpl:00939 | BP5-1 | *Cercopithifilaria japonica* | AM749263 | AM779792 | FR827942 |  |  |  |
| MIB:Zpl:00941 | BS9-1 | *C. japonica* | AM749262 | AM779793 | FR827941 | FR827915 | FR827921 | FR827917 |
| MIB:Zpl:01156 | BS6-2 | *C.japonica* | AM749261 | AM779794 | FR827943 |  |  |  |
| MIB:Zpl:00912 | S51-PB2 | *Cercopithifilaria longa* | AM749246 | AM779784 |  |  |  |  |
| MIB:Zpl:00926 | S51-PB1 | *C. longa* | AM749243 | AM779783 |  |  |  |  |
| MIB:Zpl:00930 | AG1-5 | *C. longa* | AM749245 | AM779781 |  |  |  |  |
| MIB:Zpl:00931 | AG1-10 | *C. longa* | AM749244 | AM779782 |  |  |  |  |
| MIB:Zpl:00905 | C1-A4 | *Cercopithifilaria minuta* | AM749252 | AM779785 |  |  |  |  |
| MIB:Zpl:00915 | SW1-23 | *C. minuta* | AM749253 | AM779786 |  |  |  |  |
| MIB:Zpl:00921 | G-119 | *Cercopithifilaria multicauda* | AM749255 | AM779799 |  |  |  |  |
| MIB:Zpl:00959 | 143SE | *Cercopithifilaria roussilhoni* | AM749264 | AM779798 |  |  |  |  |
| MIB:Zpl:00906 | C1-LB4 | *Cercopithifilaria shohoi* | AM749249 | AM779795 |  |  |  |  |
| MIB:Zpl:00919 | SW1-32 | *C. shohoi* | AM749250 | AM779796 |  |  |  |  |
| MIB:Zpl:00923 | SW21-170 | *C. shohoi* | AM749251 | AM779797 |  |  |  |  |
| MIB:Zpl:00901 | C1-LBB1 | *Cercopithifilaria tumidicervicata* | AM749258 | AM779787 |  |  |  |  |
| MIB:Zpl:00904 | C1-LB8 | *C. tumidicervicata* | AM749256 | AM779788 |  |  |  |  |
| MIB:Zpl:00914 | SW5-119 | *C. tumidicervicata* | AM749257 | AM779790 |  |  |  |  |
| MIB:Zpl:00918 | SW1-9 | *C. tumidicervicata* | AM749259 | AM779789 |  |  |  |  |
| MIB:Zpl:01175 | 15YU | *Dipetalonema gracile* | AM749279 | AM779824 | FR827938 |  | FR827924 | FR827918 |
| MIB:Zpl:00896 | PIS | *Dirofilaria repens* | AM749233 | AM779774 |  |  |  |  |
| MIB:Zpl:00156 | 68 CE | *Foleyella candezei* | FR823336 | FR827906 |  |  |  |  |
| MIB:Zpl:00166 | 252 JW | *Litomosa chiropterorum* | FM209527 | FM209536 |  |  |  |  |
| MIB:Zpl:00217 | 253 JW | *L. chiropterorum* | FM209528 | FM209537 |  |  |  |  |
| MIB:Zpl:00168 | 254 JW | *L. chiropterorum* | FM209529 | FM209538 |  |  |  |  |
| MIB:Zpl:00170 | 264 JW | *L. chiropterorum* | FM209532 | FM209542 |  |  |  |  |
| MIB:Zpl:00173 | 267 JW | *L. chiropterorum* | FM209534 | FM209544 |  |  |  |  |
| MIB:Zpl:00174 | 268 JW | *L. chiropterorum* |  | FM209546 |  |  |  |  |
| MIB:Zpl:00175 | 274 JW | *L. chiropterorum* |  | FM209547 |  |  |  |  |
| MIB:Zpl:01164 | 21YU | *Litomosoides sigmodontis* | AM749286 | AM779833 | FR827944 |  |  |  |
| MIB:Zpl:00175 | 44 YU | *Litomosoides taylori* | FR719324 | FR719325 | FR827945 |  |  |  |
| MIB:Zpl:01155 | 39YU | *Litomosoides yutajensis* | AM749280 | AM779825 |  |  |  |  |
| MIB:Zpl:00159 | 131 JW | *Loa loa* |  | FR827907 |  |  |  |  |
| MIB:Zpl:00160 | 132 JW | *L. loa* |  | FR827908 |  |  |  |  |
| MIB:Zpl:00902 | C1-FFL1 | *Loxodontofilaria caprini* | AM749242 | AM779822 |  |  |  |  |
| MIB:Zpl:00903 | C1-1A | *L. caprini* | AM749239 | AM779820 | FR827927 |  |  |  |
| MIB:Zpl:00928 | YG2-25 | *L. caprini* | AM749237 | AM779817 | FR827928 |  | FR827922 |  |
| MIB:Zpl:00946 | YG3-1 | *L. caprini* | AM749238 | AM779818 | FR827929 |  |  |  |
| MIB:Zpl:01149 | YG3-12 | *L. caprini* | AM749241 | AM779819 |  |  |  |  |
| MIB:Zpl:01151 | C1-SB10 | *L. caprini* | AM749240 | AM779821 |  |  |  |  |
| MIB:Zpl:00907 | S51-PB5 | *Mansonella (C.) perforata* | | AM779802 | FR827939 | FR827916 | FR827926 | FR827919 |
| MIB:Zpl:00911 | S51-PB9 | *M. (C.) perforata* | AM749265 | AM779803 |  |  |  |  |
| MIB:Zpl:00958 | 15YU | *Mansonella (T.) atelensis amazonae* | AM749278 | AM779823 | FR827940 |  |  |  |
| MIB:Zpl:01536 | 324 NB | *Mononema martini* |  | FR827912 |  |  |  |  |
| MIB:Zpl:01545 | 332 NB | *M. martini* |  | FR827911 |  |  |  |  |
| MIB:Zpl:01552 | 350 NB | *M. martini* |  | FR827914 |  |  |  |  |
| MIB:Zpl:01555 | 342 NB | *M. martini* |  | FR827913 |  |  |  |  |
| MIB:Zpl:00960 | 10YU-1 | *Ochoterenella royi* |  | AM779829 |  |  |  |  |
| MIB:Zpl:00961 | 10YU-2 | *O. royi* |  | AM779830 |  |  |  |  |
| MIB:Zpl:00913 | B61-7 | *Onchocerca dewittei japonica* | AM749266 | AM779816 | FR827933 |  |  |  |
| MIB:Zpl:00917 | B61-4 | *O. dewittei japonica* | AM749267 | AM779815 |  |  |  |  |
| MIB:Zpl:00927 | B59-1 | *O. dewittei japonica* | | AM779814 | FR827932 |  |  |  |
| MIB:Zpl:00929 | S51-9 | *Onchocerca eberhardi* | AM749268 | AM779810 | FR827937 |  |  |  |
| MIB:Zpl:00908 | S51-4 | *Onchocerca skrjabini* | AM749271 | AM779805 | FR827935 |  |  |  |
| MIB:Zpl:00909 | C1-FL5 | *O. skrjabini* | AM749273 | AM779807 | FR827934 |  | FR827925 |  |
| MIB:Zpl:00910 | S51-7 | *O. skrjabini* | AM749270 | AM779806 |  |  |  |  |
| MIB:Zpl:00916 | G30 | *O. skrjabini* | AM749272 | AM779808 |  |  |  |  |
| MIB:Zpl:00920 | SW30-26 | *O. skrjabini* | AM749274 | AM779809 |  |  |  |  |
| MIB:Zpl:00924 | S51-2 | *O. skrjabini* | AM749269 | AM779804 | FR827936 |  |  |  |
| MIB:Zpl:00932 | YG2-35 | *Onchocerca suzukii* | AM749275 | AM779811 | FR827930 |  |  |  |
| MIB:Zpl:00935 | YG2-37 | *O. suzukii* | AM749277 | AM779812 | FR827931 |  | FR827923 | FR827920 |
| MIB:Zpl:00937 | YG2-53 | *O. suzukii* | AM749276 | AM779813 |  |  |  |  |
| MIB:Zpl:01172 | 86 JW | *Onchocerca volvulus* | AM749285 | AM779855 |  |  |  |  |
| MIB:Zpl:00955 | 34YU-1 | *Piratuba scaffi* | AM749281 | AM779831 |  |  |  |  |
| MIB:Zpl:00956 | 34YU-2 | *P. scaffi* | AM749282 | AM779832 |  |  |  |  |
| MIB:Zpl:00957 | 34YU-3 | *P. scaffi* | AM749283 |  |  |  |  |  |
| MIB:Zpl:00936 | ST1 | *Setaria digitata* | AM886173 | AM779801 |  |  |  |  |
| MIB:Zpl:01159 | SET 1 | *Setaria tundra* | AM749298 | AM779848 |  |  |  |  |
| MIB:Zpl:01160 | 88YU | *Setaria* sp. 1 | AM749287 |  |  |  |  |  |
| MIB:Zpl:01162 | 85YU | *Setaria* sp. 2 | AM749289 |  |  |  |  |  |
| MIB:Zpl:01150 | 86YU | *Setaria* sp. 2 | AM749290 |  |  |  |  |  |
| MIB:Zpl:01161 | 84YU | *Setaria* sp. 3 | AM749297 |  |  |  |  |  |
| MIB:Zpl:00161 | 194 JW | *Ochoterenella* sp. 1 | FR823337 | FR827910 |  |  |  |  |
| MIB:Zpl:00162 | 193 JW | *Ochoterenella* sp. 1 | FR823338 | FR827909 |  |  |  |  |
