# Supplementary material for: New Insights into the Evolution of Wolbachia Infections in Filarial Nematodes Inferred from a Large Range of Screened Species
Source: PLoS One. 2011 Jun 22;6(6):e20843. doi: 10.1371/journal.pone.0020843 (PMC3120775; doi:10.1371/journal.pone.0020843)
Supplement: Table S3 — Wolbachia distribution in the tissues of 13 onchocercid species. +: stained; −: not stained; NA: not available because the body structure is not present. *staining shown on Figures 1– 4. (DOC) [file pone.0020843.s004.doc]

**Table S3**

*Wolbachia* distribution in the tissues of 13 onchocercid species.

| **N°** | **Species** | **Specimen Ids** | **Part** | **Hypodermis** | **Ovary** | | **Uterus** | | **Somatic germline** | **Intestine** |
| --- | --- | --- | --- | --- | --- | --- | --- | --- | --- | --- |
|  |  |  |  | **lateral chord** | **rachis** | **oocyte** | **egg or mf** | **aborted eggs** |  |  |
| 23 | *Litomosoides sigmodontis* | 32BX-1 | f* | **+** | **+** | **+** | + | NA | **-** | **-** |
|  |  | 32BX-2 | f* | **+** | **+** | **+** | **+** | NA | **-** | **-** |
| 25 | *L. yutajensis* | 102CV-1 | fa | **-** | NA | NA | NA | NA | **-** | **-** |
|  |  |  | fp | **-** | NA | **-** | NA | NA | **-** | **-** |
|  |  | 102CV-2 | fa | **-** | NA | NA | NA | NA | **-** | **-** |
|  |  |  | fc* | **-** | NA | NA | **-** | NA | **-** | **-** |
|  |  |  | fp | **-** | NA | **-** | NA | NA | **-** | **-** |
| 22 | *Litomosa chiropterorum* | 254JW-1 | fa | **-** | NA | NA | **-** | NA | **-** | **-** |
|  |  | 254JW-2 | fc | **-** | NA | NA | **-** | NA | **-** | **-** |
|  |  | 254JW-3 | fa* | **-** | NA | NA | **-** | NA | **-** | **-** |
| 13 | *Cercopithifilaria crassa* | S61-M5 | fc | **-** | NA | **-** | **-** | NA | **-** | **-** |
|  |  | S61-M4C | fp* | **-** | **-** | **-** | NA | NA | **-** | **-** |
| 14 | *C. longa* | S61-M22 | fc* | **-** | NA | NA | **-** | NA | **-** | **-** |
| 16 | *C. minuta* | C1-A5 | fp1* | **-** | NA | NA | **-** | NA | **-** | **-** |
|  |  |  | fp2 | **-** | NA | **-** | NA | NA | **-** | **-** |
| 19 | *C. shohoi* | C1-PLL3 | fp1 | **-** | **-** | NA | NA | NA | **-** | **-** |
|  |  |  | fp2* | **-** | NA | **-** | **-** | NA | **-** | **-** |
| 14 | *C. japonica* | BP5-3 | fc | **+** | NA | **+** | NA | NA | **-** | **-** |
|  |  | GB10-N6 | fp* | **+** | NA | **+** | **+** | NA | **-** | **-** |
|  |  | GB 10-3 | fa | **+** | NA | **+** | NA | NA | **-** | **-** |
|  |  |  | fc | **+** | NA | **+** | NA | NA | **-** | **-** |
|  |  |  | fp | **+** | NA | **+** | NA | NA | **-** | **-** |
| 26 | *Loxodontofilaria caprini* | C1-1 | fc | **-** | NA | NA | NA | NA | **-** | **-** |
|  |  | C1-A2 | fc* | **-** | NA | NA | **+** | NA | **-** | **-** |
| 27 | *Mansonella (Cu.) perforata* | S51-PB8 | fc* | **-** | NA | NA | **+** | **+** | **-** | **+** |
|  |  |  | fp | **-** | NA | NA | NA | NA | **-** | NA |
| 30 | *Onchocerca dewittei japonica* | B59-3 | fc | **-** | NA | NA | NA | NA | **-** | **-** |
|  |  | B59-11 | fa | **-** | NA | NA | NA | NA | **-** | **-** |
|  |  | B59**-**15 | fp* | **-** | NA | **+** | **+** | NA | **-** | **-** |
|  |  | B61-10 | fc | **-** | NA | NA | NA | NA | **-** | **-** |
| 31 | *O. eberhardi* | S51-13 | fc | **+** | NA | NA | **+** | NA | **-** | **-** |
|  |  | 1-MO9-F2 | fc* | **+** | NA | NA | **+** | NA | **-** | **-** |
|  |  | 2-MO9-F2 | fc | **+** | NA | NA | **+** | NA | **-** | **-** |
| 32 | *O. skrjabini* | S51-5 | fc* | **+** | NA | NA | NA | NA | **-** | **-** |

+ : stained; - : not stained; NA : not available because the body structure is not present. *: stainings shown on Figures 1 - 4.
